# The Cauchy Distribution in Information Theory

**DOI:** 10.3390/e25020346

**Published:** 2023-02-13

**Authors:** Sergio Verdú

**Affiliations:** Independent Researcher, Princeton, NJ 08540, USA; verdu@informationtheory.org

**Keywords:** information measures, Cauchy distribution, relative entropy, Kullback–Leibler divergence, differential entropy, Fisher’s information, entropy power inequality, *f*-divergence, Rényi divergence, mutual information, data transmission, lossy data compression

## Abstract

The Gaussian law reigns supreme in the information theory of analog random variables. This paper showcases a number of information theoretic results which find elegant counterparts for Cauchy distributions. New concepts such as that of equivalent pairs of probability measures and the strength of real-valued random variables are introduced here and shown to be of particular relevance to Cauchy distributions.

## 1. Introduction

Since the inception of information theory [1], the *Gaussian distribution* has emerged as the paramount example of a continuous random variable leading to closed-form expressions for information measures and extremality properties possessing great pedagogical value. In addition, the role of the Gaussian distribution as a ubiquitous model for analog information sources and for additive thermal noise has elevated the corresponding formulas for rate–distortion functions and capacity–cost functions to iconic status in information theory. Beyond discrete random variables, by and large, information theory textbooks confine their coverage and examples to Gaussian random variables.

The *exponential distribution* has also been shown [2] to lead to closed-form formulas for various information measures such as differential entropy, mutual information and relative entropy, as well as rate–distortion functions for Markov processes and the capacity of continuous-time timing channels with memory such as the exponential-server queue [3].

Despite its lack of moments, the *Cauchy distribution* also leads to pedagogically attractive closed-form expressions for various information measures. In addition to showcasing those, we introduce an attribute, which we refer to as the *strength* of a real-valued random variable, under which the Cauchy distribution is shown to possess optimality properties. Along with the stability of the Cauchy law, those properties result in various counterparts to the celebrated fundamental limits for memoryless Gaussian sources and channels.

To enhance readability and ease of reference, the rest of this work is organized in 120 items grouped into 17 sections, plus an appendix.

Section 2 presents the family of Cauchy random variables and their basic properties as well as multivariate generalizations, and the Rider univariate density which includes the Cauchy density as a special case and finds various information theoretic applications.

Section 3 gives closed-form expressions for the differential entropies of the univariate and multivariate densities covered in Section 2.

Introduced previously for unrelated purposes, the Shannon and η-transforms reviewed in Section 4 prove useful to derive several information theoretic results for Cauchy and related laws.

Applicable to any real-valued random variable and inspired by information theory, the central notion of *strength* is introduced in Section 5 along with its major properties. In particular, it is shown that convergence in strength is an intermediate criterion between convergence in probability and convergence in $L_{q}$, q>0, and that differential entropy is continuous with respect to the addition of independent vanishing strength noise.

Section 6 shows that, for any ρ>0 the maximal differential entropy density satisfying $E\left[\log\left(1+{\left|Z\right|}^{\rho }\right)\right]\leq \theta$ can be obtained in closed form, but its shape (not just its scale) depends on the value of θ. In particular, the Cauchy density is the solution only if ρ=2, and θ=log4. In contrast, we show that, among all the random variables with a given strength, the centered Cauchy density has maximal differential entropy, regardless of the value of the constraint. This result suggests the definition of *entropy strength* of *Z*, as the strength of a Cauchy random variable whose differential entropy is the same as that of *Z*. Modulo a factor, entropy power is the square of entropy strength. Section 6 also gives a maximal differential entropy characterization of the standard spherical Cauchy multivariate density.

Information theoretic terminology for the logarithm of the Radon–Nikodym derivative, as well as its distribution, the *relative information spectrum* is given in Section 7. The relative information spectrum for Cauchy distributions is found and shown to depend on their location and scale through a single scalar. This is a rare property, not satisfied by most common families such as Gaussian, exponential, Laplace, etc. Section 8 introduces the notion of *equivalent pairs* of probability measures, which plays an important role not only in information theory but in statistical inference. Distinguishing $P_{1}$ from $Q_{1}$ has the same fundamental limits as distinguishing $P_{2}$ from $Q_{2}$ if $\left(P_{1},Q_{1}\right)$ and $\left(P_{2},Q_{2}\right)$ are equivalent pairs. Section 9 studies the interplay between *f*-divergences and equivalent pairs. A simple formula for the *f*-divergence between Cauchy distributions results from the explicit expression for the relative information spectrum found in Section 7. These results are then used to easily derive a host of explicit expressions for $\chi^{2}$-divergence, relative entropy, total variation distance, Hellinger divergence and Rényi divergence in Section 10, Section 11, Section 12, Section 13 and Section 14, respectively.

In addition to the Fisher information matrix of the Cauchy family, Section 15 finds a counterpart of de Bruijn’s identity [4] for convolutions with scaled Cauchy random variables, instead of convolutions with scaled Gaussian random variables as in the conventional setting.

Section 16 is devoted to mutual information. The mutual information between a Cauchy random variable and its noisy version contaminated by additive independent Cauchy noise exhibits a pleasing counterpart (modulo a factor of two) with the Gaussian case, in which the signal-to-noise ratio is now given by the ratio of strengths rather than variances. With Cauchy noise, Cauchy inputs maximize mutual information under an output strength constraint. The elementary fact that an output variance constraint translates directly into an input variance constraint does not carry over to input and output strengths, and indeed we identify non-Cauchy inputs that may achieve higher mutual information than a Cauchy input with the same strength. Section 16 also considers the dual setting in which the input is Cauchy, but the additive noise need not be. Lower bounds on the mutual information, attained by Cauchy noise, are offered. However, as the bounds do not depend exclusively on the noise strength, they do not rule out the possibility that a non-Cauchy noise with identical strength may be least favorable. If distortion is measured by strength, the rate–distortion function of a Cauchy memoryless source is shown to admit (modulo a factor of two) the same rate–distortion function as the memoryless Gaussian source with mean–square distortion, replacing the source variance by its strength. Theorem 17 gives a very general continuity result for mutual information that encompasses previous such results. While convergence in probability to zero of the input to an additive-noise transformation does not imply vanishing input-output mutual information, convergence in strength does under very general conditions on the noise distribution.

Some concluding observations about generalizations and open problems are collected in Section 17, including a generalization of the notion of strength.

Those definite integrals used in the main body are collected and justified in the Appendix A.

## 2. The Cauchy Distribution and Generalizations

In probability theory, the Cauchy (also known as Lorentz and as Breit–Wigner) distribution is the prime example of a real-valued random variable none of whose moments of order one or higher exists, and as such it is not encompassed by either the law of large numbers or the central limit theorem.

1. A real-valued random variable *V* is said to be *standard Cauchy* if its probability density function is
  (1) $$\begin{array}{c} f_{V}\left(x\right)=\frac{1}{\pi }\frac{1}{x^{2}+1},\;x\in R. \end{array}$$
  Furthermore, *X* is said to be Cauchy if there exist λ≠0 and μ∈R such that X=λV+μ, in which case
  (2) $$\begin{array}{c} f_{X}\left(x\right)=\frac{\left|\lambda \right|}{\pi }\frac{1}{{\left(x-\mu \right)}^{2}+\lambda^{2}},\;x\in R, \end{array}$$
  where μ and |λ| are referred to as the *location* (or median) and *scale*, respectively, of the Cauchy distribution. If μ=0, (2) is said to be centered Cauchy.
2. Since E[max{0,V}]=E[max{0,−V}]=∞, the mean of a Cauchy random variable does not exist. Furthermore, $E[|V{|}^{q}]=\infty$ for q≥1, and the moment generating function of *V* does not exist (except, trivially, at 0). The characteristic function of the standard Cauchy random variable is
  (3) $$\begin{array}{c} E\left[e^{i\;\omega \;V}\right]=e^{-|\omega |},\;\omega \in R. \end{array}$$
3. Using (3), we can verify that a Cauchy random variable has the curious property that adding an independent copy to it has the same effect, statistically speaking, as adding an identical copy. In addition to the Gaussian and Lévy distributions, the Cauchy distribution is *stable*: a linear combination of independent copies remains in the family, and is *infinitely divisible*: it can be expressed as an *n*-fold convolution for any *n*. It follows from (3) that if $\left\{V_{1},V_{2},\ldots \right\}$ are independent, standard Cauchy, and a is a deterministic sequence with finite $\ell_{1}$-norm ${∥a∥}_{1}$, then $\sum_{i=1}^{\infty }a_{i}\;V_{i}$ has the same distribution as ${∥a∥}_{1}V$. In particular, the time average of independent identically distributed Cauchy random variables has the same distribution as any of the random variables. The families {λV,λ∈I} and {V+μ,μ∈I}, with I any interval of the real line, are some of the simplest parametrized random variables that are not an *exponential family*.
4. If Θ is uniformly distributed on $\left[-\frac{\pi }{2},\frac{\pi }{2}\right]$, then tanΘ is standard Cauchy. This follows since, in view of (1) and (A1), the standard Cauchy cumulative distribution function is
  (4) $$\begin{array}{c} F_{V}\left(x\right)=\frac{1}{2}+\frac{1}{\pi }\arctan\left(x\right),\;x\in R. \end{array}$$
  Therefore, *V* has unit semi-interquartile length. The functional inverse of (4) is the standard Cauchy *quantile function* given by
  (5) $$\begin{array}{c} Q_{V}\left(t\right)=\tan\left(\pi \left(t-\frac{1}{2}\right)\right),\;t\in \left(0,1\right). \end{array}$$
5. If $X_{1}$ and $X_{2}$ are standard Gaussian with correlation coefficient ρ∈(−1,1), then $X_{1}/X_{2}$ is Cauchy with scale $\sqrt{1-\rho^{2}}$ and location ρ. This implies that the reciprocal of a standard Cauchy random variable is also standard Cauchy.
6. Taking the cue from the Gaussian case, we say that a random vector is multivariate Cauchy if any linear combination of its components has a Cauchy distribution. Necessary and sufficient conditions for a characteristic function to be that of a multivariate Cauchy were shown by Ferguson [5]. Unfortunately, no general expression is known for the corresponding probability density function. This accounts for the fact that one aspect, in which the Cauchy distribution does not quite reach the wealth of information theoretic results attainable with the Gaussian distribution, is in the study of multivariate models of dependent random variables. Nevertheless, special cases of multivariate Cauchy distribution do admit some interesting information theoretic results as we will see below. The standard spherical multivariate Cauchy probability density function on $R^{n}$ is (e.g., [6])
  (6) $$\begin{array}{c} f_{V^{n}}\left(x\right)=\frac{\Gamma \left(\frac{n+1}{2}\right)}{\pi^{\frac{n+1}{2}}}{\left(1+{∥x∥}^{2}\right)}^{-\frac{n+1}{2}}, \end{array}$$
  where Γ(·) is the Gamma function. Therefore, $V^{n}=\left(V_{1},\ldots ,V_{n}\right)$ are exchangeable random variables. If $X_{0},X_{1},\ldots ,X_{n}$ are independent standard normal, then the vector $X_{0}^{-1}X^{n}$ has the density in (6). With the aid of (A10), we can verify that any subset of k∈{1,…,n−1} components of $V^{n}$ is distributed according to $V^{k}$. In particular, the marginals of (6) are given by (1). Generalizing (3), the characteristic function of (6) is
  (7) $$\begin{array}{c} E\left[e^{i\;t^{⊤}V^{n}}\right]=e^{-∥t∥},\;t\in R^{n}. \end{array}$$
7. In parallel to Item 1, we may generalize (6) by dropping the restriction that it be centered at the origin and allowing ellipsoidal deformation, i.e., letting $Z^{n}=\Lambda^{\frac{1}{2}}V^{n}+\mu$ with $\mu \in R^{n}$ and a positive definite n×n matrix Λ. Therefore,
  (8) $$\begin{array}{c} f_{Z^{n}}\left(x\right)=\frac{\Gamma \left(\frac{n+1}{2}\right)}{\pi^{\frac{n+1}{2}}\det^{\frac{1}{2}}\left(\Lambda \right)}{\left(1+{\left(x-\mu \right)}^{⊤}\Lambda^{-1}\left(x-\mu \right)\right)}^{-\frac{n+1}{2}}. \end{array}$$
  While $\rho^{⊤}Z^{n}$ is a Cauchy random variable for $\rho \in R^{n}-\left\{0\right\}$, (8) fails to encompass every multivariate Cauchy distribution—in particular, the important case of independent Cauchy random variables. Another reason the usefulness of the model in (8) is limited is that it is not closed under independent additions: if $V^{n}$ and ${\bar{V}}^{n}$ are independent, each distributed according to (6); then, $\Lambda^{\frac{1}{2}}V^{n}+{\bar{\Lambda }}^{\frac{1}{2}}{\bar{V}}^{n}$, while multivariate Cauchy, does not have a density of the type in (8) unless $\Lambda =\alpha \;\bar{\Lambda }$ for some α>0.
8. Another generalization of the (univariate) Cauchy distribution, which comes into play in our analysis, was introduced by Rider in 1958 [7]. With ρ>0 and βρ>1,
  (9) $$\begin{array}{cc} f_{V_{\beta ,\rho }}\left(x\right) & =\frac{\kappa_{\beta ,\rho }}{{\left(1+|x\right|}^{\rho }{)}^{\beta }},\;x\in R, \end{array}$$
  (10) $$\begin{array}{cc} \kappa_{\beta ,\rho } & =\frac{\rho \;\Gamma (\beta )}{2\;\Gamma \left(\frac{1}{\rho }\right)\Gamma \left(\beta -\frac{1}{\rho }\right)}. \end{array}$$
  In addition to the (β,ρ) parametrization in (9), we may introduce scale and location parameters by means of $\lambda V_{\beta ,\rho }+\mu$, just as we did in the Cauchy case (β,ρ)=(1,2). Another notable special case is $\sqrt{\nu }\;V_{\frac{\nu +1}{2},2}$, which is the centered Student-*t* random variable, itself equivalent to a Pearson type VII distribution.

## 3. Differential Entropy

9. The differential entropy of a Cauchy random variable is
  (11) $$\begin{array}{cc} h(\lambda V+\mu ) & =\log|\lambda |+h(V), \end{array}$$
  (12) $$\begin{array}{cc} h(V) & =-\int_{-\infty }^{\infty }f_{V}\left(t\right)\;\log f_{V}\left(t\right)\;dt=\log\left(4\pi \right), \end{array}$$
  using (A3). Throughout this paper, unless the logarithm base is explicitly shown, it can be chosen by the reader as long as it is the same on both sides of the equation. For natural logarithms, the information measure unit is the *nat*.
10. An alternative, sometimes advantageous, expression for the differential entropy of a real-valued random variable is feasible if its cumulative distribution function $F_{X}$ is continuous and strictly monotonic. Then, the quantile function is its functional inverse, i.e., $F_{X}\left(Q_{X}\left(t\right)\right)=t$ for all t∈(0,1), which implies that ${\dot{Q}}_{X}\left(t\right)f_{X}\left(Q_{X}\left(t\right)\right)=1$ for all t∈(0,1). Moreover, since *X* and $Q_{X}\left(U\right)$ with *U* uniformly distributed on [0,1] have identical distributions, we obtain
  (13) $$\begin{array}{c} h\left(X\right)=E\left[-\log f_{X}\left(X\right)\right]=E\left[-\log f_{X}\left(Q_{X}\left(U\right)\right)\right]=\int_{0}^{1}\log{\dot{Q}}_{X}\left(t\right)\;dt. \end{array}$$
  Since (4) is indeed continuous and strictly monotonic, we can verify that we recover (12) by means of (5), (13) and (A2).
11. Despite not having finite moments, an independent identically distributed sequence of Cauchy random variables $\left\{Z_{i}\right\}$ is *information stable* in the sense that
  (14) $$\begin{array}{c} \frac{1}{n}\sum_{i=1}^{n}\log f_{Z}\left(Z_{i}\right)\to -h\left(Z\right),\;a.s. \end{array}$$
  because of the strong law of large numbers.
12. With $V^{n}$ distributed according to the standard spherical multivariate Cauchy density in (6), it is shown in [8] that
  (15) $$\begin{array}{c} E\left[\log_{e}\left(1+∥V^{n}{∥}^{2}\right)\right]=\psi \left(\frac{n+1}{2}\right)+\log_{e}4+\gamma , \end{array}$$
  where γ is the Euler–Mascheroni constant and ψ(·) is the digamma function. Therefore, the differential entropy of (6) is, in nats, (see also [9])
  (16) $$\begin{array}{cc} h(V^{n}) & =\frac{n+1}{2}E\left[\log_{e}\left(1+∥V^{n}{∥}^{2}\right)\right]+\frac{n+1}{2}\log_{e}\pi -\log_{e}\Gamma \left(\frac{n+1}{2}\right) \end{array}$$
  (17) $$\begin{array}{cc} \; & =\frac{n+1}{2}\left(\log_{e}\left(4\pi \right)+\gamma +\psi \left(\frac{n+1}{2}\right)\right)-\log_{e}\Gamma \left(\frac{n+1}{2}\right), \end{array}$$
  whose growth is essentially linear with *n*: the conditional differential entropy
  $h\left(V_{n+1}|V^{n}\right)=h\left(V^{n+1}\right)-h\left(V^{n}\right)$ is monotonically decreasing with
  (18) $$\begin{array}{cc} h(V_{2}|V_{1}) & =\frac{3}{2}\left(\gamma +\psi \left(\frac{3}{2}\right)\right)+\log_{e}4=2.306... \end{array}$$
  (19) $$\begin{array}{cc} \lim_{n\to \infty }h\left(V_{n+1}|V^{n}\right) & =\frac{1}{2}\left(1+\gamma +\log_{e}\left(4\pi \right)\right)=2.054... \end{array}$$
13. By the scaling law of differential entropy and its invariance to location, we obtain
  (20) $$\begin{array}{c} h\left(\Lambda^{\frac{1}{2}}V^{n}+\mu \right)=h\left(V^{n}\right)+\frac{1}{2}\log\left|\det\left(\Lambda \right)\right|. \end{array}$$
14. Invoking (A6), we obtain a closed-form formula for the differential entropy, in nats, of the generalized Cauchy distribution (9) as
  (21) $$\begin{array}{cc} h(V_{\beta ,\rho }) & =-\log_{e}\kappa_{\beta ,\rho }+\beta \;E\left[\log_{e}\left(1+|V_{\beta ,\rho }{|}^{\rho }\right)\right] \end{array}$$
  (22) $$\begin{array}{cc} \; & =-\log_{e}\kappa_{\beta ,\rho }+\beta \;\psi \left(\beta \right)-\beta \;\psi \left(\beta -\frac{1}{\rho }\right), \end{array}$$
  with $\kappa_{\beta ,\rho }$ defined in (10).
15. The Rényi differential entropy of order α∈(0,1)∪(1,∞) of an absolutely continuous random variable with probability density function $f_{X}$ is
  (23) $$\begin{array}{c} h_{\alpha }\left(X\right)=\frac{1}{1-\alpha }\log\int f_{X}^{\alpha }\left(t\right)\;dt. \end{array}$$
  For Cauchy random variables, we obtain, with the aid of (A12),
  (24) $$\begin{array}{cc} h_{\alpha }\left(\lambda V+\mu \right) & =\log|\lambda |+h_{\alpha }\left(V\right), \end{array}$$
  (25) $$\begin{array}{cc} h_{\alpha }\left(V\right) & =\frac{\frac{1}{2}-\alpha }{1-\alpha }\log\pi +\frac{1}{1-\alpha }\log\frac{\Gamma (\alpha -\frac{1}{2})}{\Gamma (\alpha )},\;\alpha >\frac{1}{2}, \end{array}$$
  which is infinite for $\alpha \in (0,\frac{1}{2}]$, converges to log(4π) (cf. (12)) as α→1, and to logπ, the reciprocal of the mode height, as α→∞.
16. Invoking (A13), the Rényi differential entropy of order $\alpha \in \left(\frac{1}{\beta \rho },1\right)\cup \left(1,\infty \right)$ of the generalized Cauchy distribution (9) is
  (26) $$\begin{array}{c} h_{\alpha }\left(V_{\beta ,\rho }\right)=\frac{\alpha }{1-\alpha }\log\kappa_{\beta ,\rho }+\frac{1}{1-\alpha }\log\frac{2\;\Gamma \left(\beta \alpha -\frac{1}{\rho }\right)\Gamma \left(\frac{1}{\rho }\right)}{\rho \;\Gamma (\beta \alpha )}. \end{array}$$

## 4. The Shannon- and η-Transforms

In this section, we recall the definitions of two notions introduced in [10] for the unrelated purpose of expressing the asymptotic singular value distribution of large random matrices.

17. The Shannon transform of a nonnegative random variable *X* is the function $V_{X}:\left[0,\infty \right)\to \left[0,\infty \right)$, defined by
  (27) $$\begin{array}{cc} V_{X}\left(\theta \right) & =E\left[\log_{e}\left(1+\theta \;X\right)\right]. \end{array}$$
  Unless $V_{X}\left(\theta \right)=\infty$ for all θ>0 (e.g., if *X* has the log-Cauchy density $\frac{1}{\pi x}\frac{1}{1+\log^{2}x},x>0$), or $V_{X}\left(\theta \right)=0$, θ≥0, (which occurs if X=0 a.s.), the Shannon transform is a strictly concave continuous function from $V_{X}\left(0\right)=0$, which grows without bound as θ→∞.
18. If *V* is standard Cauchy, then (A4) results in
  (28) $$\begin{array}{cc} V_{V^{2}}\left(\theta^{2}\right) & =2\log_{e}\left(1+|\theta |\right), \end{array}$$
  and the handy relationship
  (29) $$\begin{array}{c} E\left[\log\left(\beta^{2}+\lambda^{2}V^{2}\right)\right]=2\log\left(|\beta |+|\lambda |\right). \end{array}$$
19. For the distribution in (9) with (β,ρ)=(2,2), (A7) results in
  (30) $$\begin{array}{cc} V_{V_{2,2}^{2}}\left(\theta^{2}\right) & =2\log_{e}\left(1+|\theta |\right)-\frac{2|\theta |}{1+|\theta |}. \end{array}$$
20. The η-transform $\eta_{X}:\left[0,\infty \right)\to \left(0,1\right]$ of a non-negative random variable is defined as the function
  (31) $$\begin{array}{c} \eta_{X}\left(\theta \right)=E\left[\frac{1}{1+\theta X}\right]=1-\theta \;{\dot{V}}_{X}\left(\theta \right), \end{array}$$
  which is intimately related to the Cauchy–Stieltjes transform [11]. For example,
  (32) $$\begin{array}{cc} \eta_{V^{2}}\left(\theta^{2}\right) & =\frac{1}{1+|\theta |}, \end{array}$$
  (33) $$\begin{array}{cc} \eta_{V_{2,2}^{2}}\left(\theta^{2}\right) & =\frac{1+2|\theta |}{{\left(1+|\theta |\right)}^{2}}. \end{array}$$

## 5. Strength

The purpose of this section is to introduce an attribute which is particularly useful to compare random variables that do not have finite moments.

21. The *strength* ς(Z)∈[0,+∞] of a real-valued random variable *Z* is defined as
  (34) $$\begin{array}{c} \varsigma \left(Z\right)=\inf\left\{\varsigma >0:E\left[\log\left(1+\frac{Z^{2}}{\varsigma^{2}}\right)\right]\leq \log4\right\}. \end{array}$$
  It follows that the only random variable with zero strength is Z=0, almost surely. If the inequality in (34) is not satisfied for any ς>0, then ς(Z)=∞. Otherwise, ς(Z) is the unique positive solution ς>0 to
  (35) $$\begin{array}{c} E\left[\log\left(1+\frac{Z^{2}}{\varsigma^{2}}\right)\right]=\log4. \end{array}$$
  If ς(Z)≤ς, then (35) holds with ≤.
22. The set of probability measures whose strength is upper bounded by a given finite nonnegative constant,
  (36) $$\begin{array}{c} A_{\varsigma }=\left\{P_{Z}:\varsigma \left(Z\right)\leq \varsigma \right\}, \end{array}$$
  is convex: The set $A_{0}$ is a singleton as seen in Item 21, while, for 0<ς<∞, we can express (36) as
  (37) $$\begin{array}{c} A_{\varsigma }=\left\{P_{Z}:E\left[\log\left(1+\frac{Z^{2}}{\varsigma^{2}}\right)\right]\leq \log4\right\}. \end{array}$$
  Therefore, if $P_{Z_{0}}\in A_{\varsigma }$ and $P_{Z_{1}}\in A_{\varsigma }$, we must have $\alpha P_{Z_{1}}+\left(1-\alpha \right)P_{Z_{0}}\in A_{\varsigma }$.
23. The peculiar constant in the definition of strength is chosen so that if *V* is standard Cauchy, then its strength is ς(V)=1 because, in view of (29),
  (38) $$\begin{array}{c} E\left[\log\left(1+V^{2}\right)\right]=\log4. \end{array}$$
24. If Z=k∈R, a.s., then its strength is
  (39) $$\begin{array}{c} \varsigma \left(Z\right)=\frac{\left|k\right|}{\sqrt{3}}. \end{array}$$
25. The left side of (35) is the *Shannon transform* of $Z^{2}$ evaluated at $\varsigma^{-2}$, which is continuous in $\varsigma^{2}$. If ς(Z)∈(0,∞) then, (35) can be written as
  (40) $$\begin{array}{c} \varsigma^{2}\left(Z\right)=\frac{1}{V_{Z^{2}}^{-1}\left(\log_{e}4\right)}, \end{array}$$
  where, on the right side, we have denoted the functional inverse of the Shannon transform. Clearly, the square root of the right side of (40) cannot be expressed as the expectation with respect to *Z* of any b:R→R that does not depend on $P_{Z}$. Nevertheless, thanks to (37), (36) can be expressed as
  (41) $$\begin{array}{c} A_{\varsigma }=\left\{P_{Z}:E\left[b_{\varsigma^{2}}\left(Z\right)\right]\leq 1\right\},\;\;with\;b_{\varsigma^{2}}\left(x\right)=\log_{4}\left(1+\frac{x^{2}}{\varsigma^{2}}\right). \end{array}$$
26.
  **Theorem** **1.** *The strength of a real-valued random variable satisfies the following properties:*
  *(a)*  (42) $$\begin{array}{c} \varsigma (\lambda Z)=|\lambda |\;\varsigma (Z). \end{array}$$
  *(b)*  (43) $$\begin{array}{c} \varsigma^{2}\left(Z\right)\leq \frac{1}{3}\;E\left[Z^{2}\right], \end{array}$$
    *with equality if and only if |Z| is deterministic.*
  *(c)*  *If 0<q<2, and ${∥Z∥}_{q}=E^{\frac{1}{q}}{\left[|Z\right|}^{q}]<\infty$, then*
    (44) $$\begin{array}{c} \varsigma \left(Z\right)\leq \kappa_{q}^{\frac{1}{q}}\;{∥Z∥}_{q},\;with\;\;\kappa_{q}=\max_{x>0}\frac{\log_{4}\left(1+x^{2}\right)}{x^{q}}. \end{array}$$
  *(d)*  *If V is standard Cauchy, independent of X, then ς(X+V) is the solution to*
    (45) $$\begin{array}{c} V_{X^{2}}\left({\left(\varsigma +1\right)}^{-2}\right)=2\log\frac{2}{1+\varsigma^{-1}}, \end{array}$$
    *if it exists, otherwise, ς(X+V)=∞. Moreover, ≤ holds in (45) if ς(X+V)≤ς.*
  *(e)*  (46) $$\begin{array}{c} 2\log\left(2\min\{1,\varsigma (Z)\}\right)\leq E\left[\log\left(1+Z^{2}\right)\right]\leq 2\log\left(2\max\{1,\varsigma (Z)\}\right). \end{array}$$
  *(f)*  *If 0<ς(Z)<∞, then*
    (47) $$\begin{array}{c} h(Z)=\log(4\pi \;\varsigma (Z))-D(Z\;∥\;\varsigma (Z)V), \end{array}$$
    *where V is standard Cauchy, and D(X∥Y) stands for the relative entropy with reference probability measure $P_{Y}$ and dominated measure $P_{X}$.*
  *(g)*  (48) $$\begin{array}{c} h\left(Z\right)<\infty \;\;⟸\;\;\varsigma \left(Z\right)<\infty \;\;⟺\;\;E\left[\log\left(1+Z^{2}\right)\right]<\infty . \end{array}$$
  *(h)*  *If V is standard Cauchy, then*
    (49) $$\begin{array}{c} \varsigma (Z)<\infty \;\;and\;\;h(Z)\in R\;\;⟺\;\;D(Z\;∥\;\lambda \;V)<\infty ,\;forall\;\;\lambda >0. \end{array}$$
  *(i)*  *The finiteness of strength is sufficient for the finiteness of the entropy of the integer part of the random variable, i.e.,*
    H(⌊Z⌋)=∞⟹ς(Z)=∞.
  *(j)*  *If $Z_{n}\to Z$ in $L_{q}$ for any q∈(0,1], then $\varsigma \left(Z_{n}\right)\to \varsigma \left(Z\right)$.*
  *(k)*  (50) $$\begin{array}{c} Z_{n}\to 0\;\;i.p.\;\;⟸\;\;E\left[\log\left(1+Z_{n}^{2}\right)\right]\to 0\;\;⟺\;\;\varsigma \left(Z_{n}\right)\to 0. \end{array}$$
  *(l)*  *If $\varsigma (X_{n})\to 0$, then $\varsigma \left(Z+X_{n}\right)\to \varsigma \left(Z\right)$.*
  *(m)*  *If $\varsigma (X_{n})\to 0$, ς(Z)<∞ and Z is independent of $X_{n}$, then $h\left(Z+X_{n}\right)\to h\left(Z\right)$.*
  **Proof.**  For the first three properties, it is clear that they are satisfied if ς(Z)=0, i.e., Z=0 almost surely.
  (a) If $\varsigma^{2}\in \left(0,\infty \right)$ is the solution to (35), then $\lambda^{2}\;\varsigma^{2}$ is a solution to (35) with λZ taking the role of *Z*. If (35) has no solution, neither does its version in which λZ takes the role of *Z*.
  (b) Jensen’s inequality applied to the left side of (35) results in $3\varsigma^{2}\leq E\left[Z^{2}\right]$. The strict concavity of log(1+t) implies that equality holds if and only if $Z^{2}$ is deterministic. If (35) has no solution, the same reasoning implies that $E[Z^{2}]=\infty$.
  (c) First, it is easy to check that, for q∈(0,2), the function $f_{q}:\left(0,\infty \right)\to \left(0,\infty \right)$ given by $f_{q}\left(t\right)=t^{-q}\log_{4}\left(1+t^{2}\right)$ attains its maximum $\kappa_{q}$ at a unique point. Assume ς(Z)∈(0,∞). Since $\kappa_{q}\;t^{q}\geq \log_{4}\left(1+t^{2}\right)$ for all t>0, letting t=|Z|/ς(Z) and taking expectations, (35) (choosing 4 as the logarithm base) results in
    (51) $$\begin{array}{c} \frac{\kappa_{q}}{\varsigma^{q}\left(Z\right)}E\left[{\left|Z\right|}^{q}\right]\geq 1, \end{array}$$
    which is the same as (44). If ς(Z)=∞, then $\infty =E\left[\log\left(1+Z^{2}\right)\right]\leq \kappa_{q}{\;E[|Z|}^{q}]$.
  (d) Invoking (A4) with $\alpha^{2}=\varsigma^{2}+x^{2}$ and $\left|\sin\beta \right|=\frac{\varsigma }{\sqrt{x^{2}+\varsigma^{2}}}$, we obtain
    (52) $$\begin{array}{cc} E\left[\log\left(1+\frac{{\left(x+V\right)}^{2}}{\varsigma^{2}}\right)\right] & =\log\left(\frac{{\left(1+\varsigma \right)}^{2}+x^{2}}{\varsigma^{2}}\right) \end{array}$$
    (53) $$\begin{array}{cc} \; & =\log\left(1+\frac{x^{2}}{{\left(1+\varsigma \right)}^{2}}\right)-2\log\frac{\varsigma }{\varsigma +1}. \end{array}$$
    Substituting *x* by *X* and averaging over *X*, the result follows from the definition of strength.
  (e) The result holds trivially if either ς(Z)=0 or ς(Z)=∞. Otherwise, we simply rewrite (35) as
    (54) $$\begin{array}{c} 2\log\left(2\varsigma \left(Z\right)\right)=E\left[\log\left(\varsigma^{2}\left(Z\right)+Z^{2}\right)\right], \end{array}$$
    and upper/lower bound the right side by $E\left[\log\left(1+Z^{2}\right)\right]$.
  (f) (55) $$\begin{array}{cc} D(Z\;∥\;\varsigma (Z)V)\; & =-h\left(Z\right)+\log\left(\varsigma \left(Z\right)\;\pi \right)+E\left[\log\left(1+\frac{Z^{2}}{\varsigma^{2}\left(Z\right)}\right)\right] \end{array}$$
    (56) $$\begin{array}{cc} \; & =\log(4\pi \;\varsigma (Z))-h(Z), \end{array}$$
    where (55) and (56) follow from (2) and (35), respectively.
  (g)
    - If ς(Z)<∞, then $E\left[\log\left(1+Z^{2}\right)\right]<\infty$ and h(Z)<∞ follow from (46) and (47), respectively.
    - If $E\left[\log(1+Z^{2})\right]<\infty$, the dominated convergence theorem implies
      (57) $$\begin{array}{c} \lim_{\varsigma \to \infty }E\left[\log\left(1+{\left(\frac{Z}{\varsigma }\right)}^{2}\right)\right]=0. \end{array}$$
      Excluding the case Z=0 a.s. for which both $E\left[\log(1+Z^{2})\right]$ and ς(Z) are zero, we have
      (58) $$\begin{array}{c} \lim_{\varsigma ↓0}E\left[\log\left(1+{\left(\frac{Z}{\varsigma }\right)}^{2}\right)\right]=\lim_{\varsigma ↓0}V_{Z^{2}}\left(\frac{1}{\varsigma^{2}}\right)=\infty . \end{array}$$
      Since (35) is continuous in ς, it must have a finite solution in view of (57) and (58).
  (h) It is sufficient to assume λ=1 for the condition on the right of (49) because the condition on the left holds if and only if it holds for αZ, for any α>0 and D(αZ∥αV)=D(Z∥V). If h(Z)<∞, then
    (59) $$\begin{array}{c} D\left(Z\;∥\;V\right)=-h\left(Z\right)+\log\pi +E\left[\log\left(1+Z^{2}\right)\right], \end{array}$$
    which is finite unless either h(Z)=−∞ or $E[\log\left(1+Z^{2}\right)]=\infty$. This establishes ⟹ in view of (48). To establish ⟸, it is enough to show that
    (60) $$\begin{array}{c} D\left(Z\;∥\;V\right)<\infty ⟹E\left[\log\left(1+Z^{2}\right)\right]<\infty , \end{array}$$
    in view of (48) and the fact that, according to (59), h(Z)>−∞ if both D(Z∥V) and $E\left[\log\left(1+Z^{2}\right)\right]$ are finite. To show (60), we invoke the following variational representation of relative entropy (first noted by Kullback [12] for absolutely continuous random variables): If $P_{Z}\ll P_{V}$, then
    (61) $$\begin{array}{c} D\left(Z\;∥\;V\right)=\max_{Q:Q\ll P_{V}}E\left[\log\frac{dQ}{dP_{V}}\left(Z\right)\right], \end{array}$$
    attained only at $Q=P_{Z}$. Let *Q* be the absolutely continuous random variable with probability density function
    (62) $$\begin{array}{c} q\left(x\right)=\frac{\log_{e}2}{4|x|\log_{e}^{2}\left|x\right|}\;1\left\{|x|\geq 2\right\}+\frac{1}{8}1\left\{|x|<2\right\}. \end{array}$$
    Then,
    (63) $$\begin{array}{cc} \infty & >D\left(Z\;∥\;V\right)>E\left[\log\frac{q(Z)}{f_{V}\left(Z\right)}\right]\\ \; & =E\left[1\left\{|Z|\geq 2\right\}\left(\log\frac{\pi \log_{e}2}{4}+\log\left(1+Z^{2}\right)-\log\left(\left|Z\right|\log_{e}^{2}\left|Z\right|\right)\right)\right] \end{array}$$
    (64) $$\begin{array}{cc} \; & \;+E\left[1\left\{|Z|<2\right\}\left(\log\frac{\pi }{8}+\log\left(1+Z^{2}\right)\right)\right] \end{array}$$
    (65) $$\begin{array}{cc} \; & >\frac{1}{5}E\left[1\left\{Z\geq 2\right\}\log\left(1+Z^{2}\right)\right]+\log\frac{5\pi }{8} \end{array}$$
    (66) $$\begin{array}{cc} \; & \geq \frac{1}{5}E\left[\log\left(1+Z^{2}\right)\right]+\frac{4}{5}\log5-\log\frac{8}{\pi }, \end{array}$$
    where (65) holds since
    (67) $$\begin{array}{c} \frac{4}{5}\log\left(1+x^{2}\right)\geq -\log\left(\pi \log_{e}2\right)+2\log\log_{e}\left|x\right|+\log|x|,\;|x|>2. \end{array}$$
  (i) (68) $$\begin{array}{cc} \varsigma (Z)<\infty & ⟹\;E\left[\log(1+Z^{2})\right]<\infty \end{array}$$
    (69) $$\begin{array}{cc} \; & ⟹\;E\left[\log(1+|Z|)\right]<\infty \end{array}$$
    (70) $$\begin{array}{cc} \; & ⟹\;H(\lfloor Z\rfloor )<\infty , \end{array}$$
    where (68)–(70) follow from (48), $\log\left(1+x^{2}\right)\leq 2\log\left(1+|x|\right)$, and p. 3743 in [13], respectively.
  (j) If ς(Z)=0, then Z=0 a.e., and the result follows from (44). For all $\left(x,z\right)\in R^{2}$,
    (71) $$\begin{array}{cc} \left|\log_{e}\left(\frac{1+{\left(x+z\right)}^{2}}{1+z^{2}}\right)\right| & \leq \log_{e}\left(1+\frac{1}{2}(x^{2}+\left|x\right|\sqrt{4+x^{2}})\right) \end{array}$$
    (72) $$\begin{array}{cc} \; & \leq \frac{2}{q}{\left|x\right|}^{q}, \end{array}$$
    where (71) follows by maximizing the left side over z∈R. Denote the difference between the right side and the left side of (72) by $f_{q}\left(x\right)$, an even function which satisfies $f_{q}\left(0\right)=0$, and
    (73) $$\begin{array}{c} {\dot{f}}_{q}\left(x\right)=2x^{q-1}-\frac{2}{\sqrt{4+x^{2}}}>0,\;x>0,\;0<q\leq 1. \end{array}$$
    Therefore, (72) follows. Assuming 0<ς(Z)<∞, we have
    (74) $$\begin{array}{cc} \left|E\left[\log(1+Z_{n}^{2})\right]-E\left[\log(1+Z^{2})\right]\right|\; & \leq E\left[\left|\log\left(1+Z_{n}^{2}\right)-\log\left(1+Z^{2}\right)\right|\right] \end{array}$$
    (75) $$\begin{array}{cc} \; & \leq \frac{2}{q}E\left[|Z_{n}{-Z|}^{q}\right]\;\log e. \end{array}$$
    Now, because of the scaling property in (42), we may assume without loss of generality that ς(Z)=1. Thus, (74) and (75) result in
    (76) $$\begin{array}{c} \left|E\left[\log(1+Z_{n}^{2})\right]-\log4\right|\leq \frac{2}{q}E\left[|Z_{n}{-Z|}^{q}\right]\;\log e, \end{array}$$
    which requires that $\varsigma (Z_{n})\to 1$, since, by assumption, the right side vanishes. Assume now that ς(Z)=∞, and therefore, $E\left[\log(1+Z^{2})\right]=\infty$. Inequality (75) remains valid in this case, implying that, as soon as the right side is finite (which it must be for all sufficiently large *n*), $E\left[\log(1+Z_{n}^{2})\right]=\infty$, and therefore, $\varsigma (Z_{n})=\infty$ in view of (48).
  (k)
1st ⟸
     For any ϵ>0, Markov’s inequality results in
      (77) $$\begin{array}{c} P[|Z_{n}\left|>\epsilon \right]=P\left[\log\left(1+Z_{n}^{2}\right)>\log\left(1+\epsilon^{2}\right)\right]\leq \frac{E\left[\log\left(1+Z_{n}^{2}\right)\right]}{\log\left(1+\epsilon^{2}\right)}. \end{array}$$
    ⟹ First, we show that, for any α>0, we have
      (78) $$\begin{array}{c} E\left[\log\left(1+Z_{n}^{2}\right)\right]\to 0\;\;⟹E\left[\log\left(1+\alpha \;Z_{n}^{2}\right)\right]\to 0. \end{array}$$
      The case 0<α<1 is trivial. The case α>1 follows because $E\left[\log\left(1+Z_{n}^{2}\right)\right]$→0 implies
      (79) $$\begin{array}{c} E\left[\log\left(1+\alpha \;Z_{n}^{2}\right)\right]=E\left[\log\left(1+\left(\alpha -1\right)\;Z_{n}^{2}\right)\right], \end{array}$$
      where ≥ is obvious, and ≤ holds because
      (80) $$\begin{array}{cc} \log\left(1+\alpha \;t^{2}\right) & =\log\left(1+t^{2}\right)+\log\left(1+\left(\alpha -1\right)\frac{t^{2}}{1+t^{2}}\right) \end{array}$$
      (81) $$\begin{array}{cc} \; & \leq \log\left(1+t^{2}\right)+\log\left(1+\left(\alpha -1\right)\;t^{2}\right). \end{array}$$
      If $\varsigma (Z_{n})=\infty$ infinitely often, so is $E\left[\log\left(1+Z_{n}^{2}\right)\right]$ in view of (48). Assume that $lim sup\varsigma \left(Z_{n}\right)=\varsigma \in \left(0,\infty \right]$, and $\varsigma (Z_{n})$ is finite for all sufficiently large. Then, there is a subsequence such that $\varsigma (Z_{n_{i}})↛$, and
      (82) $$\begin{array}{c} \log4=E\left[\log\left(1+{\left(\frac{Z_{n_{i}}}{\varsigma (Z_{n_{i}})}\right)}^{2}\right)\right]\leq E\left[\log\left(1+{\left(\frac{Z_{n_{i}}}{\lambda }\right)}^{2}\right)\right], \end{array}$$
      for all sufficiently large *i* and λ<ς. Consequently, (78) implies that $E\left[\log\left(1+Z_{n}^{2}\right)\right]\neg \to 0$.
2nd ⟸
   Suppose that $E\left[\log\left(1+Z_{n}^{2}\right)\right]↛0$. Therefore, there is a subsequence along which $E\left[\log\left(1+Z_{n_{i}}^{2}\right)\right]>\eta >0$. If η≥log4, then $\varsigma (Z_{n_{i}})>1$ along the subsequence. Because of the continuity of the Shannon transform and the fact that it grows without bound as its argument goes to infinity (Item 25), if 0<η<log4, we can find 1<α<∞ such that $E\left[\log\left(1+\alpha Z_{n_{i}}^{2}\right)\right]>\log4$, which implies $\varsigma \left(Z_{n_{i}}\right)>\alpha^{-1/2}$. Therefore, $\varsigma (Z_{n})↛0$ as we wanted to show.
  (l) We start by showing that
    (83) $$\begin{array}{c} E\left[\log\left(1+X_{n}^{2}\right)\right]\to 0\;\;⟺\;\;E\left[f(X_{n})\right]\to 0, \end{array}$$
    where we have denoted the right side of (71) with arbitrary logarithm base by f(x). Since $\dot{f}\left(x\right)=\frac{2\log e}{\sqrt{4+x^{2}}}$, it is easy to verify that
    (84) $$\begin{array}{c} 0\leq f\left(x\right)-\log\left(1+x^{2}\right)\leq \log\frac{4}{3},\;x\in R, \end{array}$$
    where the lower and upper bounds are attained uniquely at x=0 and $\left|x\right|=\frac{1}{\sqrt{2}}$, respectively. The lower bound results in ⟸ in (83). To show ⟹, decompose, for arbitrary ϵ>0,
    (85) $$\begin{array}{cc} E\left[f(X_{n})\right] & =E\left[f\left(X_{n}\right)1\{|X_{n}\left|<\epsilon \right\}\right]+E\left[f\left(X_{n}\right)1\{|X_{n}\left|\geq \epsilon \right\}\right] \end{array}$$
    (86) $$\begin{array}{cc} \; & \leq f\left(\epsilon \right)+E\left[f\left(X_{n}\right)1\{|X_{n}\left|\geq \epsilon \right\}\right] \end{array}$$
    (87) $$\begin{array}{cc} \; & \leq f\left(\epsilon \right)+A_{\epsilon }\;E\left[\log\left(1+X_{n}^{2}\right)1\{|X_{n}\left|\geq \epsilon \right\}\right] \end{array}$$
    (88) $$\begin{array}{cc} \; & \leq f\left(\epsilon \right)+A_{\epsilon }\;\epsilon^{3}, \end{array}$$
    where
    (89) $$\begin{array}{cc} A_{\epsilon } & =1+\frac{\log\frac{4}{3}}{\log(1+\epsilon^{2})}, \end{array}$$
    (87) holds from the upper bound in (84), and the fact that (89) is decreasing in ϵ, and (88) holds for all sufficiently large *n* if $E\left[\log\left(1+X_{n}^{2}\right)\right]\to 0$. Since the right side of (88) goes to 0 as ϵ→0, (83) is established. Assume 0<ς(Z)<∞. From the linearity property (42), we have $\varsigma \left(Z+X_{n}\right)=\varsigma \left(Z\right)\cdot \varsigma \left(\bar{Z}+{\bar{X}}_{n}\right)$ with $\bar{Z}=\varsigma^{-1}\left(Z\right)\;Z$ and ${\bar{X}}_{n}=\varsigma^{-1}\left(Z\right)\;X_{n}$ which satisfies $\varsigma ({\bar{X}}_{n})\to 0$. Therefore, we may restrict attention to ς(Z)=1 without loss of generality. Following (71) and (74), and abbreviating $Z_{n}=Z+X_{n}$, we obtain
    (90) $$\begin{array}{cc} \left|E\left[\log(1+Z_{n}^{2})\right]-\log4\right|\; & \leq E\left[\left|\log\left(1+Z_{n}^{2}\right)-\log\left(1+Z^{2}\right)\right|\right] \end{array}$$
    (91) $$\begin{array}{cc} \; & \leq E\left[f(X_{n})\right]. \end{array}$$
    Thus, the desired result follows in view of (50) and (83). To handle the case ς(Z)=∞, we use the same reasoning as in the proof of (i) since (83) remains valid in that case.
  (m) If ς(Z)=0, then Z=0 a.s., h(Z)=−∞ and $h(X_{n})\to -\infty$ in view of Part (f). Assume henceforth that ς(Z)>0. Since $h\left(Z+X_{n}\right)\geq h\left(Z\right)$, it suffices to show
    (92) $$\begin{array}{c} \underset{n\to \infty }{lim sup}h\left(X_{n}+Z\right)\leq h\left(Z\right). \end{array}$$
    Under the assumptions, Part (l) guarantees that
    (93) $$\begin{array}{c} \varsigma \left(X_{n}+Z\right)\to \varsigma \left(Z\right). \end{array}$$
    If *V* is a standard Cauchy random variable, then $\varsigma \left(Z+X_{n}\right)V\to \varsigma \left(Z\right)V$ in distribution as the characteristic function converges: $e^{-\varsigma (Z+X_{n})|t|}\to e^{-\varsigma (Z)|t|}$ for all *t*. Analogously, according to Part (k), $Z+X_{n}\overset{D}{⟶}Z$ since $X_{n}\to 0$ in probability. Since the strength of $X_{n}+Z$ is finite for all sufficiently large *n*, we may invoke (47) to express, for those *n*,
    (94) $$\begin{array}{cc} h\left(X_{n}+Z\right)-h\left(Z\right) & =\log\frac{\varsigma (Z+X_{n})}{\varsigma (Z)}\\ \; & \;-D\left(Z+X_{n}\;∥\;\varsigma \left(Z+X_{n}\right)\;V\right)+D\left(Z\;∥\;\varsigma \left(Z\right)\;V\right). \end{array}$$
    The lower semicontinuity of relative entropy under weak convergence (which, in turn, is a corollary to the Donsker–Varadhan [14,15] variational representation of relative entropy) results in
    (95) $$\begin{array}{c} \underset{n\to \infty }{lim inf}D\left(Z+X_{n}\;∥\;\varsigma \left(Z+X_{n}\right)V\right)\geq D\left(Z\;∥\;\varsigma \left(Z\right)V\right), \end{array}$$
    because $Z+X_{n}\overset{D}{⟶}Z$ and $\varsigma \left(Z+X_{n}\right)V\overset{D}{⟶}\varsigma \left(Z\right)V$. Therefore, (92) follows from (94) and (95).
       □
27. In view of (42) and Item 23, ς(λV)=|λ| if *V* is standard Cauchy. Furthermore, if $X_{1}$ and $X_{2}$ are centered independent Cauchy random variables, then their sum is centered Cauchy with
  (96) $$\begin{array}{c} \varsigma \left(X_{1}+X_{2}\right)=\varsigma \left(X_{1}\right)+\varsigma \left(X_{2}\right). \end{array}$$
  More generally, it follows from Theorem 1-(d) that, if $X_{1}$ is centered Cauchy, and (96) holds for $X_{2}=\alpha X$ and all α∈R, then *X* must be centered Cauchy. Invoking (52), we obtain
  (97) $$\begin{array}{c} \varsigma \left(\lambda V+\mu \right)=\frac{\left|\lambda \right|}{3}+\frac{1}{3}\sqrt{4\lambda^{2}+3\mu^{2}}, \end{array}$$
  which is also valid for λ=0 as we saw in Item 24.
28. If *X* is standard Gaussian, then $\varsigma^{2}\left(X\right)=0.171085\cdots$, and $\varsigma^{2}\left(\sigma X\right)=\sigma^{2}\varsigma^{2}\left(X\right)$. Therefore, if $X_{1}$ and $X_{2}$ are zero-mean independent Gaussian random variables, then
  (98) $$\begin{array}{c} \varsigma^{2}\left(X_{1}+X_{2}\right)=\varsigma^{2}\left(X_{1}\right)+\varsigma^{2}\left(X_{2}\right). \end{array}$$
  Thus, in this case, $\varsigma \left(X_{1}+X_{2}\right)<\varsigma \left(X_{1}\right)+\varsigma \left(X_{2}\right)$.
29. It follows from Theorem 1-(d) that, with *X* independent of standard Cauchy *V*, we obtain ς(X+V)>ς(X)+ς(V) whenever *X* is such that
  (99) $$\begin{array}{c} V_{X^{2}}\left({\left(2+\varsigma \left(X\right)\right)}^{-2}\right)>2\;\log_{e}\left(1+\frac{\varsigma (X)}{2+\varsigma (X)}\right). \end{array}$$
  An example is the heavy-tailed probability density function
  (100) $$\begin{array}{c} f_{X}\left(x\right)=\frac{1}{\pi }\frac{\log_{4}\left(1+x^{2}\right)}{1+x^{2}}, \end{array}$$
  for which 7.0158…=ς(X+V)>ς(X)+ς(V)=6.8457….
30. Using (A8), we can verify that, if *X* is zero-mean uniform with variance $\sigma^{2}$, then
  (101) $$\begin{array}{c} \varsigma^{2}\left(X\right)=\frac{3}{c^{2}}\sigma^{2}=0.221618\ldots \sigma^{2}, \end{array}$$
  where *c* is the solution to $\log_{e}\left(1+c^{2}\right)+\frac{2}{c}\arctan\left(c\right)=2+\log_{e}4$.
31. We say that $Z_{n}\to 0$ in strength if $\varsigma (Z_{n})\to 0$. Parts (j) and (k) of Theorem 1 show that this convergence criterion is intermediate between the traditional in probability and $L_{q}$ criteria. It is not equivalent to either one: If
  (102) $$\begin{array}{c} Z_{n}=\left\{\begin{array}{cc} 0, & \mathrm{with}\;\mathrm{probability}\;1-\frac{1}{n};\\ 2^{n}, & \mathrm{with}\;\mathrm{probability}\;\frac{1}{n}, \end{array}\right. \end{array}$$
  then $\varsigma (Z_{n})\to 1$, while $Z_{n}\to 0$ in probability. If, instead, $Z_{n}={\frac{3}{2}}^{n}$, with probability $\frac{1}{n}$, then $Z_{n}\to 0$ in strength, but not in $L_{q}$ for any 0<q.
32. The assumption in Theorem 1-(m) that $X_{n}\to 0$ in strength cannot be weakened to convergence in probability. Suppose that $X_{n}$ is absolutely continuous with probability density function
  (103) $$\begin{array}{c} f_{X_{n}}\left(t\right)=\left\{\begin{array}{cc} n-1, & t\in \left[0,\frac{1}{n}\right];\\ 0, & t\in \left(-\infty ,0\right)\cup \left(\frac{1}{n},2\right);\\ \frac{1}{n}\frac{\log_{e}2}{t\log_{e}^{2}t}, & t\in [2,\infty ). \end{array}\right. \end{array}$$
  We have $X_{n}\to 0$ in probability since, regardless of how small ϵ>0, $P\left[X_{n}>\epsilon \right]=\frac{1}{n}$ for all $n\geq \frac{1}{\epsilon }$. Furthermore,
  (104) $$\begin{array}{c} h\left(X_{n}+Z\right)\geq h\left(X_{n}\right)=\infty , \end{array}$$
  because (103) is the mixture of a uniform and an infinite differential entropy probability density function, and differential entropy is concave. We conclude that $h\left(X_{n}+Z\right)↛h\left(Z\right)$, since h(Z)<∞.
33. The following result on the continuity of differential entropy is shown in [16]: if *X* and *Z* are independent, E[|Z|]<∞ and E[|X|]<∞, then
  (105) $$\begin{array}{c} \lim_{\epsilon ↓0}h\left(\epsilon X+Z\right)=h\left(Z\right). \end{array}$$
  This result is weaker than Theorem 1-(m) because finite first absolute moment implies finite strength as we saw in (44), and ϵX→0 in $L_{1}$ if ϵ→0, and therefore, it vanishes in strength too.
34. If *Z* and *V* are centered and standard Cauchy, respectively, then $\min_{\lambda }D\left(Z\;∥\;\lambda V\right)$ is achieved by λ=ς(Z). Otherwise, in general, this does not hold. Since $D\left(Z\;∥\;\lambda V\right)=V_{Z^{2}}\left(\lambda^{-2}\right)-h\left(Z\right)+\log_{e}\left(\pi \lambda \right)$, the minimum is attained at the solution to
  (106) $$\begin{array}{c} \eta_{Z^{2}}\left(\frac{1}{\lambda_{*}^{2}}\right)=\frac{1}{2}, \end{array}$$
  where we have used the η-transform in (31). If $Z=V_{2,2}$, recalling (32), (106), results in $\lambda_{*}=\sqrt{2}-1$, while $\varsigma (V_{2,2})=0.302\ldots$.
35. Using (28) and the concavity of log(1+x), we can verify that
  (107) $$\begin{array}{c} \varsigma \left(X_{\alpha }\right)\leq \alpha \varsigma \left(X_{1}\right)+\left(1-\alpha \right)\varsigma \left(X_{0}\right),\;X_{\alpha }∼\alpha P_{X_{1}}+\left(1-\alpha \right)P_{X_{0}}, \end{array}$$
  if $X_{0}$ and $X_{1}$ are centered Cauchy, or, more generally, if $X_{0}=\lambda_{0}X$, $X_{1}=\lambda_{1}X$ and $V_{X^{2}}\left(\theta^{2}\right)$ is concave on θ. Not only is this property not satisfied if X=1 but (107) need not hold in that case, as we can verify numerically for α=0.1, $\lambda_{1}=1$ and $\lambda_{0}>20$.

## 6. Maximization of Differential Entropy

36. Among random variables with a given second moment (resp. first absolute moment), differential entropy is maximized by the zero-mean Gaussian (resp. Laplace) distribution. More generally, among random variables with a given *p*-absolute moment μ, differential entropy is maximized by the parameter-*p* Subbotin (or generalized normal) distribution with *p*-absolute moment μ [17]
  (108) $$\begin{array}{c} f_{X}\left(x\right)=\frac{p^{1-\frac{1}{p}}}{2\;\Gamma \left(\frac{1}{p}\right)\;\mu^{\frac{1}{p}}}e^{-\frac{{\left|x\right|}^{p}}{p\;\mu }},\;x\in R. \end{array}$$
  Among nonnegative random variables with a given mean, differential entropy is maximized by the exponential distribution. In those well-known solutions, the cost function is an affine function of the negative logarithm of the maximal differential entropy probability density function. Is there a cost function such that, among all random variables with a given expected cost, the Cauchy distribution is the maximal differential entropy solution? To answer this question, we adopt a more general viewpoint. Consider the following result, whose special case ρ=2 was solved in [18] using convex optimization:
  **Theorem** **2.** *Fix ρ>0 and θ>0.*
  (109) $$\begin{array}{c} \max_{Z:E\left[\log_{e}\left(1+{\left|Z\right|}^{\rho }\right)\right]\leq \theta }h\left(Z\right)=h\left(V_{\beta ,\rho }\right), \end{array}$$
  *where $V_{\beta ,\rho }$ is defined in (9), the right side of (109) is given in (22), and $\beta >\rho^{-1}$ is the solution to*
  (110) $$\begin{array}{c} \theta =\psi \left(\beta \right)-\psi \left(\beta -\frac{1}{\rho }\right). \end{array}$$
  *Therefore, the standard Cauchy distribution is the maximal differential entropy distribution provided that ρ=2 and $\theta =\log_{e}4.$*
  **Proof.** 
  (a) For every ρ>0 and θ>0, there is a unique $\beta >\rho^{-1}$ that satisfies (110) because the function of β on the right side is strictly monotonically decreasing, grows without bound as $\beta ↓\frac{1}{\rho }$, and goes to zero as β→∞.
  (b) For any *Z* which satisfies $E\left[\log_{e}\left(1+{\left|Z\right|}^{\rho }\right)\right]\leq \theta$, its relative entropy, in nats, with respect to $V_{\beta ,\rho }$ is
    (111) $$\begin{array}{cc} D(Z\;∥\;V_{\beta ,\rho }) & =-h\left(Z\right)-\log_{e}\kappa_{\beta ,\rho }+\beta \;E\left[\log_{e}\left(1+{\left|Z\right|}^{\rho }\right)\right] \end{array}$$
    (112) $$\begin{array}{cc} \; & \leq -h\left(Z\right)-\log_{e}\kappa_{\beta ,\rho }+\beta \;\theta \end{array}$$
    (113) $$\begin{array}{cc} \; & =-h\left(Z\right)-\log_{e}\kappa_{\beta ,\rho }+\beta \;\psi \left(\beta \right)-\beta \;\psi \left(\beta -\frac{1}{\rho }\right) \end{array}$$
    (114) $$\begin{array}{cc} \; & =h\left(V_{\beta ,\rho }\right)-h\left(Z\right), \end{array}$$
    where (113) and (114) follow from (110) and (22), respectively. Since relative entropy is nonnegative, and zero only if both measures are identical, not only does (2) hold but any random variable other than $V_{\beta ,\rho }$ achieves strictly lower differential entropy.
       □
37. An unfortunate consequence stemming from Theorem 2 is that, while we were able to find out a cost function such that the Cauchy distribution is the maximal differential entropy distribution under an average cost constraint, this holds only for a specific value of the constraint. This behavior is quite different from the classical cases discussed in Item 36 for which the solution is, modulo scale, the same regardless of the value of the cost constraint. As we see next, this deficiency is overcome by the notion of strength introduced in Section 5.
38.
  **Theorem** **3.** *Strength constraint.* *The differential entropy of a real-valued random variable with strength ς(Z) is upper bounded by*
(115) $$\begin{array}{c} h\left(Z\right)\leq \log\left(4\pi \;\varsigma (Z)\right). \end{array}$$
*If 0<ς(Z)<∞, equality holds if and only if Z has a centered Cauchy density, i.e., Z=λV for some λ>0.*
  **Proof.** 
  (a) If *Z* is not an absolutely continuous random variable, or more generally, h(Z)=−∞ such as in the case ς(Z)=0 in which Z=0 with probability one, then (115) is trivially satisfied.
  (b) If 0<ς(Z)<∞ and h(Z)>−∞, then we invoke (47) to conclude that not only does (115) hold, but it is satisfied with equality if and only if Z=ς(Z)V.
     □
39. The entropy power of a random variable *Z* is the variance of a Gaussian random variable whose differential entropy is h(Z), i.e.,
  (116) $$\begin{array}{c} N\left(Z\right)=\frac{1}{2\pi e}\exp\left(2\;h(Z)\right). \end{array}$$
  While the power of a Cauchy random variable is infinite, its entropy power is given by
  (117) $$\begin{array}{c} N\left(\lambda V+\mu \right)=\frac{1}{2\pi e}\exp\left(2\;h(\lambda V+\mu )\right)=\frac{8\;\pi \;\lambda^{2}}{e}. \end{array}$$
  In the same spirit as the definition of entropy power, Theorem 3 suggests the definition of $N_{C}\left(Z\right)$, the *entropy strength* of *Z*, as the strength of a centered Cauchy random variable whose differential entropy is h(Z), i.e., $h\left(Z\right)=h\left(N_{C}\left(Z\right)V\right)$. Therefore,
  (118) $$\begin{array}{cc} N_{C}\left(Z\right) & =\frac{1}{4\pi }\exp\left(h(Z)\right) \end{array}$$
  (119) $$\begin{array}{cc} \; & =\varsigma \left(Z\right)\;\exp\left(-D\left(Z\;∥\;\varsigma (Z)V\right)\right) \end{array}$$
  (120) $$\begin{array}{cc} \; & \leq \varsigma (Z), \end{array}$$
  where (119) follows from (56), and (120) holds with equality if and only if *Z* is centered Cauchy. Note that, for all $\left(\alpha ,\mu \right)\in R^{2}$,
  (121) $$\begin{array}{c} N_{C}\left(\alpha \;Z+\mu \right)=\left|\alpha \right|\;N_{C}\left(Z\right). \end{array}$$
  Comparing (116) and (118), we see that entropy power is simply a scaled version of the entropy strength squared,
  (122) $$\begin{array}{c} N\left(Z\right)=\frac{8\pi }{e}N_{C}^{2}\left(Z\right). \end{array}$$
  The entropy power inequality (e.g., [19,20]) states that, if $X_{1}$ and $X_{2}$ are independent real-valued random variables, then
  (123) $$\begin{array}{c} N\left(X_{1}+X_{2}\right)\geq N\left(X_{1}\right)+N\left(X_{2}\right), \end{array}$$
  regardless of whether they have moments. According to (122), we may rewrite the entropy power inequality (123) replacing each entropy power by the corresponding squared entropy strength. Therefore, the squared entropy strength of the sum of independent random variables satisfies
  (124) $$\begin{array}{c} N_{C}^{2}\left(X_{1}+X_{2}\right)\geq N_{C}^{2}\left(X_{1}\right)+N_{C}^{2}\left(X_{2}\right). \end{array}$$
  It is well-known that equality holds in (123), and hence (124), if and only if both random variables are Gaussian. Indeed, if $X_{1}$ and $X_{2}$ are centered Cauchy with respective strengths $\varsigma_{1}>0$ and $\varsigma_{2}>0$, then (124) becomes ${\left(\varsigma_{1}+\varsigma_{2}\right)}^{2}>\varsigma_{1}^{2}+\varsigma_{2}^{2}$.
40. Theorem 3 implies that any random variable with infinite differential entropy has infinite strength. There are indeed random variables with finite differential entropy and infinite strength. For example, let Z∈[2,∞) be an absolutely continuous random variable with probability density function
  (125) $$\begin{array}{c} f_{Z}\left(t\right)=\left\{\begin{array}{cc} 0.473991...\log_{e}^{-2}n, & t\in \left[n,n+\frac{1}{n}\right],\;\;n\in \left\{2,3,\ldots \right\};\\ 0, & \mathrm{elsewhere}. \end{array}\right. \end{array}$$
  Then, h(Z)=1.99258... nats, while the entropy of the quantized version as well as the strength satisfy H(⌊Z⌋)=∞=ς(Z).
41. With the same approach, we may generalize Theorem 3 to encompass the full slew of the generalized Cauchy distributions in (9). To that end, fix ρ>0 and define the (ρ,θ)-*strength* of a random variable as
  (126) $$\begin{array}{c} \varsigma_{\rho ,\theta }\left(Z\right)=\inf\left\{\varsigma >0:E\left[\log_{e}\left(1+{\left|\frac{Z}{\varsigma }\right|}^{\rho }\right)\right]\leq \theta \right\}. \end{array}$$
  Therefore, $\varsigma_{\rho ,\theta }\left(Z\right)=\varsigma \left(Z\right)$ for $\left(\rho ,\theta \right)=\left(2,\log_{e}4\right)$, and if (β,ρ,θ) satisfy (110), then $\varsigma_{\rho ,\theta }\left(V_{\beta ,\rho }\right)=1$. As in Item 25, if $\varsigma_{\rho ,\theta }\left(Z\right)\in \left(0,\infty \right)$, we have
  (127) $$\begin{array}{c} \varsigma_{\rho ,\theta }^{\rho }\left(Z\right)=\frac{1}{V_{{\left|Z\right|}^{\rho }}^{-1}\left(\theta \right)}. \end{array}$$
42.
  **Theorem** **4.** *Generalized strength constraint.* *Fix ρ>0 and θ>0. The differential entropy of a real-valued random variable with (ρ,θ)-strength $\varsigma_{\rho ,\theta }\left(Z\right)$ is upper bounded by*
(128) $$\begin{array}{c} h\left(Z\right)\leq \log\left(\varsigma_{\rho ,\theta }\left(Z\right)\right)+h\left(V_{\beta ,\rho }\right), \end{array}$$
  *where β is given by the solution to (110), $V_{\beta ,\rho }$ has the generalized Cauchy density (9), and $h(V_{\beta ,\rho })$ is given in (21). If $\varsigma_{\rho ,\theta }\left(Z\right)<\infty$, equality holds if and only if Z is a constant times $V_{\beta ,\rho }$.*
  **Proof.**  As with Theorem 3, in the proof, we may assume $0<\varsigma_{\rho ,\theta }\left(Z\right)<\infty$ to avoid trivialities. Then,
  (129) $$\begin{array}{c} E\left[\log_{e}\left(1+{\left|\frac{Z}{\varsigma_{\rho ,\theta }\left(Z\right)}\right|}^{\rho }\right)\right]=\theta , \end{array}$$
  and, in nats,
  (130) $$\begin{array}{cc} D\left(\frac{Z}{\varsigma_{\rho ,\theta }\left(Z\right)}\;∥\;V_{\beta ,\rho }\right)\; & =-h\left(Z\right)-\log_{e}\frac{\kappa_{\beta ,\rho }}{\varsigma_{\rho ,\theta }\left(Z\right)}+\beta \;E\left[\log_{e}\left(1+{\left|\frac{Z}{\varsigma_{\rho ,\theta }\left(Z\right)}\right|}^{\rho }\right)\right] \end{array}$$
  (131) $$\begin{array}{cc} \; & =-h\left(Z\right)-\log_{e}\frac{\kappa_{\beta ,\rho }}{\varsigma_{\rho ,\theta }\left(Z\right)}+\beta \;\theta \end{array}$$
  (132) $$\begin{array}{cc} \; & =-h\left(Z\right)-\log_{e}\frac{\kappa_{\beta ,\rho }}{\varsigma_{\rho ,\theta }\left(Z\right)}+\beta \;\psi \left(\beta \right)-\beta \;\psi \left(\beta -\frac{1}{\rho }\right) \end{array}$$
  (133) $$\begin{array}{cc} \; & =-h\left(Z\right)+\log_{e}\left(\varsigma_{\rho ,\theta }\left(Z\right)\right)+h\left(V_{\beta ,\rho }\right), \end{array}$$
  where (130), (131), (132), and (133) follow from (9), (129), (110), and (22), respectively.    □
43. In the multivariate case, we may find a simple upper bound on differential entropy based on the strength of the norm of the random vector.
  **Theorem** **5.** *The differential entropy of a random vector $Z^{n}$ is upper bounded by*
(134) $$\begin{array}{c} h\left(Z^{n}\right)\leq n\;\log\left(\varsigma (∥Z^{n}∥)\right)+\frac{n+1}{2}\log\left(4\pi \right)-\log\Gamma \left(\frac{n+1}{2}\right). \end{array}$$
  **Proof.**  As in the proof of Theorem 3, we may assume that $0<\varsigma (∥Z^{n}∥)<\infty$. As usual, $V^{n}$ denotes the standard spherical multivariate Cauchy density in (6). Since for α≠0, $f_{\alpha V^{n}}\left(x^{n}\right)={\left|\alpha \right|}^{-n}f_{V^{n}}\left(\alpha^{-1}x^{n}\right)$, we have
  (135) $$\begin{array}{cc} \; & D(Z^{n}\;∥\;\varsigma (∥Z^{n}∥)\;V^{n})=-h\left(Z^{n}\right)-E\left[\log f_{\varsigma (∥Z^{n}∥)V^{n}}\left(Z^{n}\right)\right] \end{array}$$
  (136) $$\begin{array}{cc} \; & =-h\left(Z^{n}\right)+n\;\log\left(\varsigma (∥Z^{n}∥)\right)-\log\frac{\Gamma \left(\frac{n+1}{2}\right)}{\pi^{\frac{n+1}{2}}}+\frac{n+1}{2}E\left[\log\left(1+\frac{∥Z^{n}{∥}^{2}}{\varsigma^{2}(∥Z^{n}∥)}\right)\right] \end{array}$$
  (137) $$\begin{array}{cc} \; & =-h\left(Z^{n}\right)+n\;\log\left(\varsigma (∥Z^{n}∥)\right)-\log\Gamma \left(\frac{n+1}{2}\right)+\frac{n+1}{2}\log\left(4\pi \right), \end{array}$$
  where (136) and (137) follow from (6) and the definition of strength, respectively.    □
  For n=1, Theorem 5 becomes the bound in (115). For n=2,3,…, the right side of (15) is greater than $\log_{e}4$, and, therefore, $\varsigma (∥Z^{n}∥)>1$. Consequently, in the multivariate case, there is no $Z^{n}$ such that (134) is tight.
44. To obtain a full generalization of Theorem 3 in the multivariate case, it is advisable to define the strength of a random *n*-vector as
  (138) $$\begin{array}{cc} \varsigma (Z^{n})\; & =\inf\left\{\varsigma >0:-E\left[\log f_{V^{n}}\left(\varsigma^{-1}Z^{n}\right)\right]\leq h\left(V^{n}\right)\right\} \end{array}$$
  (139) $$\begin{array}{cc} \; & =\varsigma_{2,\theta_{n}}(∥Z^{n}∥) \end{array}$$
  for $\theta_{n}=\psi \left(\frac{n+1}{2}\right)+\gamma +\log_{e}4$. To verify (139), note (15)–(17). Notice that $\varsigma (\lambda V^{n})=|\lambda |$ and for n=1, (138) is equal to (34). The following result provides a maximal differential entropy characterization of the standard spherical multivariate Cauchy density.
  **Theorem** **6.** *Let $V^{n}$ have the standard multivariate Cauchy density (6), Then,*
(140) $$\begin{array}{c} h\left(Z^{n}\right)\leq n\;\log\varsigma \left(Z^{n}\right)+h\left(V^{n}\right), \end{array}$$
  *where $h(V^{n})$ is given in (17). If $0<\varsigma (Z^{n})<\infty$, equality holds in (140) if and only if $Z^{n}=\lambda V^{n}$ for some λ≠0.*
  **Proof.**  Assume $0<\varsigma (Z^{n})<\infty$. Then,
  (141) $$\begin{array}{cc} D\left(\frac{Z^{n}}{\varsigma (Z^{n})}\;∥\;V^{n}\right)\; & =-h\left(Z^{n}\right)+n\log\varsigma \left(Z^{n}\right)-E\left[\log f_{V^{n}}\left(\varsigma^{-1}\left(Z^{n}\right)Z^{n}\right)\right] \end{array}$$
  (142) $$\begin{array}{cc} \; & =-h\left(Z^{n}\right)+n\log\varsigma \left(Z^{n}\right)+h\left(V^{n}\right) \end{array}$$
  in view of (138). Hence, the difference between right and left sides of (140) is equal to zero if and only if $Z^{n}=\lambda V^{n}$ for some λ≠0; otherwise, it is positive.    □

## 7. Relative Information

45. For probability measures *P* and *Q* on the same measurable space (A,F), such that P≪Q, the logarithm of their Radon–Nikodym derivative is the *relative information* denoted by
  (143) $$\begin{array}{c} {ı}_{P∥Q}\left(x\right)=\log\frac{dP}{dQ}\left(x\right). \end{array}$$
46. As usual, we may employ the notation ${ı}_{X∥Y}\left(x\right)$ to denote ${ı}_{P_{X}∥P_{Y}}\left(x\right)$. The distributions of the random variables ${ı}_{X∥Y}\left(X\right)$ and ${ı}_{X∥Y}\left(Y\right)$ are referred to as *relative information spectra* (e.g., [21]). It can be shown that there is a one-to-one correspondence between the cumulative distributions of ${ı}_{X∥Y}\left(X\right)$ and ${ı}_{X∥Y}\left(Y\right)$. For example, if they are absolutely continuous random variables with respective probability density functions $f_{X∥Y}$ and ${\bar{f}}_{X∥Y}$, then
  (144) $$\begin{array}{c} f_{X∥Y}\left(\alpha \right)=\exp\left(\alpha \right)\;{\bar{f}}_{X∥Y}\left(\alpha \right),\;\alpha \in R. \end{array}$$
  Obviously, the distributions of ${ı}_{X∥Y}\left(X\right)$ and $\frac{dP_{X}}{dP_{Y}}\left(X\right)$ determine each other. One caveat is that relative information may take the value −∞. It can be shown that
  (145) $$\begin{array}{cc} P[{ı}_{X∥Y}\left(X\right)=-\infty ] & =0, \end{array}$$
  (146) $$\begin{array}{cc} P[{ı}_{X∥Y}\left(Y\right)=-\infty ] & =1-E\left[\exp(-{ı}_{X∥Y}\left(X\right))\right]. \end{array}$$
47. The information spectra determine all measures of the distance between the respective probability measures of interest (e.g., [22,23]), including *f*-divergences and Rényi divergences. For example, the *relative entropy* (or Kullback–Leibler divergence) of the dominated measure *P* with respect to the reference measure *Q* is the average of the relative information when the argument is distributed according to *P*, i.e., $D\left(X∥Y\right)=E[{ı}_{X∥Y}\left(X\right)]$. If P¬≪Q, then D(P∥Q)=∞.
48. The information spectra also determine the fundamental trade-off in hypothesis testing. Let $\alpha_{\nu }\left(P_{1},P_{0}\right)$ denote the minimal probability of deciding $P_{0}$ when $P_{1}$ is true subject to the constraint that the probability of deciding $P_{1}$ when $P_{0}$ is true is no larger than ν. A consequence of the Neyman–Pearson lemma is
  (147) $$\begin{array}{c} \alpha_{\nu }\left(P_{1},P_{0}\right)=\min_{\gamma \in R}\left\{P\left[{ı}_{P_{1}∥P_{0}}\left(Y_{1}\right)\leq \gamma \right]-\exp\left(\gamma \right)\left(\nu -P\left[{ı}_{P_{1}∥P_{0}}\left(Y_{0}\right)>\gamma \right]\right)\right\}, \end{array}$$
  where $Y_{0}∼P_{0}$ and $Y_{1}∼P_{1}$.
49. Cauchy distributions are absolutely continuous with respect to each other and, in view of (2),
  (148) $$\begin{array}{c} {ı}_{\lambda_{1}V+\mu_{1}∥\lambda_{0}V+\mu_{0}}\left(x\right)=\log\frac{|\lambda_{1}|}{|\lambda_{0}|}+\log\frac{{\left(x-\mu_{0}\right)}^{2}+\lambda_{0}^{2}}{{\left(x-\mu_{1}\right)}^{2}+\lambda_{1}^{2}}. \end{array}$$
50. The following result, proved in Item 58, shows that the relative information spectrum corresponding to Cauchy distributions with respective scale/locations $\left(\lambda_{1},\mu_{1}\right)$ and $\left(\lambda_{0},\mu_{0}\right)$ depends on the four parameters through the single scalar
  (149) $$\begin{array}{c} \zeta \left(\lambda_{1},\mu_{1},\lambda_{0},\mu_{0}\right)=\frac{\lambda_{1}^{2}+\lambda_{0}^{2}+{\left(\mu_{1}-\mu_{0}\right)}^{2}}{2|\lambda_{0}\lambda_{1}|}\geq 1, \end{array}$$
  where equality holds if and only if $\left(\lambda_{1},\mu_{1}\right)=\left(\lambda_{0},\mu_{0}\right)$.
  **Theorem** **7.** *Suppose that $\lambda_{1}\lambda_{0}\neq 0$, and V is standard Cauchy. Denote*
(150) $$\begin{array}{c} Z=\frac{dP_{\lambda_{1}V+\mu_{1}}}{dP_{\lambda_{0}V+\mu_{0}}}\left(\lambda_{1}V+\mu_{1}\right). \end{array}$$
  *Then,*
  *(a)* 
(151) $$\begin{array}{c} E\left[Z\right]=\zeta \left(\lambda_{1},\mu_{1},\lambda_{0},\mu_{0}\right), \end{array}$$
  *(b)*  *Z has the same distribution as the random variable*
(152) $$\begin{array}{c} \zeta +\sqrt{\zeta^{2}-1}\;\cos\Theta , \end{array}$$
*where Θ is uniformly distributed on [−π,π] and $\zeta =\zeta (\lambda_{1},\mu_{1},\lambda_{0},\mu_{0})$. Therefore, the probability density function of Z is*
(153) $$\begin{array}{c} f_{Z}\left(z\right)=\frac{1}{\pi }\frac{1}{\sqrt{\zeta^{2}-1-{\left(z-\zeta \right)}^{2}}}, \end{array}$$
*on the interval $0<\zeta -\sqrt{\zeta^{2}-1}<z<\zeta +\sqrt{\zeta^{2}-1}$.*
51. The indefinite integral (e.g., see 2.261 in [24])
  (154) $$\begin{array}{c} \int \frac{dx}{\sqrt{2\zeta x-x^{2}-1}}=\arcsin\frac{x-\zeta }{\sqrt{\zeta^{2}-1}} \end{array}$$
  results, with $X_{i}=\lambda_{i}V+\mu_{i}$, i=0,1, in
  (155) $$\begin{array}{c} P\left[{ı}_{X_{1}∥X_{0}}\left(X_{1}\right)\leq \log t\right]=\left\{\begin{array}{cc} 1, & \zeta +\sqrt{\zeta^{2}-1}\leq t;\\ \frac{1}{2}+\frac{1}{\pi }\arcsin\frac{t-\zeta }{\sqrt{\zeta^{2}-1}}, & \zeta -\sqrt{\zeta^{2}-1}<t<\zeta +\sqrt{\zeta^{2}-1};\\ 0, & 0<t\leq \zeta -\sqrt{\zeta^{2}-1}. \end{array}\right. \end{array}$$
52. For future use, note that the endpoints of the support of (153) are their respective reciprocals. Furthermore,
  (156) $$\begin{array}{c} f_{Z}\left(\frac{1}{z}\right)=z\;f_{Z}\left(z\right), \end{array}$$
  which implies
  (157) $$\begin{array}{c} f_{\frac{1}{Z}}\left(z\right)=\frac{1}{z}\;f_{Z}\left(z\right). \end{array}$$

## 8. Equivalent Pairs of Probability Measures

53. Suppose that $P_{1}$ and $Q_{1}$ are probability measures on $\left(A_{1},F_{1}\right)$ such that $P_{1}\ll Q_{1}$ and $P_{2}$ and $Q_{2}$ are probability measures on $\left(A_{2},F_{2}\right)$ such that $P_{2}\ll Q_{2}$. We say that $\left(P_{1},Q_{1}\right)$ and $\left(P_{2},Q_{2}\right)$ are *equivalent pairs*, and write $\left(P_{1},Q_{1}\right)\equiv \left(P_{2},Q_{2}\right)$, if the cumulative distribution functions of ${ı}_{P_{1}∥Q_{1}}\left(X_{1}\right)$ and ${ı}_{P_{2}∥Q_{2}}\left(X_{2}\right)$ are identical with $X_{1}∼P_{1}$ and $X_{2}∼P_{2}$. Naturally, ≡ is an equivalence relationship. Because of the one-to-one correspondence indicated in Item 46, the definition of equivalent pairs does not change if we require equality of the information spectra under the dominated measure, i.e., that ${ı}_{P_{1}∥Q_{1}}\left(Y_{1}\right)$ and ${ı}_{P_{2}∥Q_{2}}\left(Y_{2}\right)$ be equally distributed $Y_{1}∼Q_{1}$ and $Y_{2}∼Q_{2}$. Obviously, the requirement that the information spectra coincide is the same as requiring that the distributions of $\frac{dP_{1}}{dQ_{1}}\left(Y_{1}\right)$ and $\frac{dP_{2}}{dQ_{2}}\left(Y_{2}\right)$ are equal. As in Item 46, we also employ the notation $\left(X_{1},Y_{1}\right)\equiv \left(X_{2},Y_{2}\right)$ to indicate $\left(P_{1},Q_{1}\right)\equiv \left(P_{2},Q_{2}\right)$ if $X_{1}∼P_{1}$, $X_{2}∼P_{2}$, $Y_{1}∼Q_{1}$, and $Y_{2}∼Q_{2}$.
54. Suppose that the output probability measures of a certain (random or deterministic) transformation are $Q_{0}$ and $Q_{1}$ when the input is distributed according to $P_{0}$ and $P_{1}$, respectively. If $\left(P_{0},P_{1}\right)\equiv \left(Q_{0},Q_{1}\right)$, then the transformation is a sufficient statistic for deciding between $P_{0}$ and $P_{1}$ (i.e., the case of a binary parameter).
55. If (A,F) is a measurable space on which the probability measures $P_{X_{1}}\ll P_{X_{2}}$ are defined, and ϕ:A→B is a (F,G)-measurable *injective* function, then $P_{\phi (X_{1})}\ll P_{\phi (X_{2})}$ are probability measures on (B,G) and
  (158) $$\begin{array}{c} {ı}_{X_{1}∥X_{2}}\left(x\right)={ı}_{\phi \left(X_{1}\right)∥\phi \left(X_{2}\right)}\left(\phi (x)\right). \end{array}$$
  Consequently, $\left(X_{1},X_{2}\right)\equiv \left(\phi \left(X_{1}\right),\phi \left(X_{2}\right)\right)$.
56. The most important special case of Item 55 is an affine transformation of an arbitrary real-valued random variable *X*, which enables the reduction of four-parameter problems into two-parameter problems: for all $\left(\lambda_{2},\mu_{1},\mu_{2}\right)\in R^{3}$ and $\lambda_{1}\neq 0$,
  (159) $$\begin{array}{c} \left(\lambda_{1}X+\mu_{1},\lambda_{2}X+\mu_{2}\right)\equiv \left(X,\lambda X+\mu \right), \end{array}$$
  with
  (160) $$\begin{array}{c} \lambda =\frac{\lambda_{2}}{\lambda_{1}}\;\;and\;\;\mu =\frac{\mu_{2}-\mu_{1}}{\lambda_{1}}, \end{array}$$
  by choosing the affine function $\phi \left(x\right)=\frac{x-\mu_{1}}{\lambda_{1}}$.
57.
  **Theorem** **8.** *If $X^{n}\in R^{n}$ is an even random vector, i.e., $P_{X^{n}}=P_{-X^{n}}$, then*
(161) $$\begin{array}{c} \left(X^{n}+\mu_{1},X^{n}+\mu_{2}\right)\equiv \left(X^{n}+\mu_{3},X^{n}+\mu_{4}\right), \end{array}$$
  *whenever $|\mu_{1}-\mu_{2}\left|=\right|\mu_{3}-\mu_{4}|$.*
  **Proof.** 
  (a) If $\mu_{1}-\mu_{2}=\mu_{3}-\mu_{4}$, then (161) holds even if $X^{n}$ is not even because the function x−μ is injective, in particular, with $\mu =\mu_{3}-\mu_{1}=\mu_{4}-\mu_{2}$.
  (b) If $\mu_{1}-\mu_{2}=\mu_{4}-\mu_{3}$, then
    (162) $$\begin{array}{cc} (X^{n}+\mu_{1},X^{n}+\mu_{2})\; & \equiv (X^{n},X^{n}+\mu_{2}-\mu_{1}) \end{array}$$
    (163) $$\begin{array}{cc} \; & \equiv (X^{n},X^{n}+\mu_{3}-\mu_{4}) \end{array}$$
    (164) $$\begin{array}{cc} \; & \equiv (-X^{n}+\mu_{3}-\mu_{4},-X^{n}) \end{array}$$
    (165) $$\begin{array}{cc} \; & \equiv (X^{n}+\mu_{3}-\mu_{4},X^{n}) \end{array}$$
    (166) $$\begin{array}{cc} \; & \equiv (X^{n}+\mu_{3},X^{n}+\mu_{4}), \end{array}$$
    where (162) and (166) follow from Part (a), (164) follows because $-x+\mu_{3}-\mu_{4}$ is injective, and (165) holds because $X^{n}$ is even.
       □
58. We now proceed to prove Theorem 7.
  **Proof.**  Since λV and −λV have identical distributions, we may assume for convenience that $\lambda_{1}>0$ and $\lambda_{0}>0$. Furthermore, capitalizing on Item 56, we may assume $\lambda_{1}=1$, $\mu_{1}=0$, $\lambda_{0}=\lambda$, and $\mu_{0}=\mu$, and then recover the general result letting $\lambda =\frac{\lambda_{0}}{\lambda_{1}}$ and $\mu =\frac{\mu_{0}-\mu_{1}}{\lambda_{1}}$. Invoking (A9) and (A10), we have
  (167) $$\begin{array}{cc} E\left[\frac{dP_{V}}{dP_{\lambda V+\mu }}\left(V\right)\right] & =\frac{1}{\lambda }E\left[\frac{{\left(V-\mu \right)}^{2}+\lambda^{2}}{V^{2}+1}\right] \end{array}$$
  (168) $$\begin{array}{cc} \; & =\frac{1}{\pi \;\lambda }\int_{-\infty }^{\infty }\frac{{\left(t-\mu \right)}^{2}+\lambda^{2}}{{\left(t^{2}+1\right)}^{2}}\;dt \end{array}$$
  (169) $$\begin{array}{cc} \; & =\frac{\lambda^{2}+\mu^{2}+1}{2\;\lambda }, \end{array}$$
  and we can verify that we recover (151) through the aforementioned substitution. Once we have obtained the expectation of $Z=\frac{dP_{V}}{dP_{\lambda V+\mu }}\left(V\right)$, we proceed to determine its distribution. Denoting the right side of (169) by ζ, we have
  (170) $$\begin{array}{cc} Z-E[Z] & =\frac{1}{\lambda }\frac{\lambda^{2}+{\left(V-\mu \right)}^{2}}{1+V^{2}}-\zeta \end{array}$$
  (171) $$\begin{array}{cc} \; & =\frac{1}{2\lambda }\frac{\left(1-\lambda^{2}-\mu^{2}\right)\left(V^{2}-1\right)-4\mu V}{1+V^{2}} \end{array}$$
  (172) $$\begin{array}{cc} \; & =\frac{1}{2\lambda }\left(1-\lambda^{2}-\mu^{2}\right)\left(\sin^{2}\Theta -\cos^{2}\Theta \right)-4\mu \sin\Theta \cos\Theta \end{array}$$
  (173) $$\begin{array}{cc} \; & =\frac{1}{2\lambda }\left((\lambda^{2}+\mu^{2}-1)\cos2\Theta -2\mu \sin2\Theta \right) \end{array}$$
  (174) $$\begin{array}{cc} \; & =\frac{1}{2\lambda }\sqrt{{\left(\lambda^{2}+\mu^{2}-1\right)}^{2}+4\mu^{2}}\;\cos\left(2\Theta +\phi_{\lambda ,\mu }\right) \end{array}$$
  (175) $$\begin{array}{cc} \; & =\sqrt{\zeta^{2}-1}\;\cos\left(2\Theta +\phi_{\lambda ,\mu }\right), \end{array}$$
  where Θ is uniformly distributed on [−π,π]. We have substituted V=tanΘ (see Item 4) in (172), and invoked elementary trigonometric identities in (173) and (174). Since the phase in (175) does not affect it, the distribution of *Z* is indeed as claimed in (152), and (153) follows because the probability density function of cosΘ is
  (176) $$\begin{array}{c} f_{\cos\Theta }\left(t\right)=\frac{1}{\pi }\frac{1}{\sqrt{1-t^{2}}},\;\left|t\right|<1. \end{array}$$
     □
59. In general, it need not hold that (X,Y)≡(Y,X)—for example, if *X* and *Y* are zero-mean Gaussian with different variances. However, the class of scalar Cauchy distributions does satisfy this property since the result of Theorem 7 is invariant to swapping $\lambda_{1}↔\lambda_{0}$ and $\mu_{1}↔\mu_{0}$. More generally, Theorem 7 implies that, if $\lambda_{1}\lambda_{0}\gamma_{1}\gamma_{0}\neq 0$, then
  (177) $$\begin{array}{cc} \left(\lambda_{1}V+\mu_{1},\lambda_{0}V+\mu_{0}\right) & \equiv (\gamma_{1}V+\nu_{1},\gamma_{0}V+\nu_{0})\\ \; & ⇕\;\\ \frac{\lambda_{1}^{2}+\lambda_{0}^{2}+{\left(\mu_{1}-\mu_{0}\right)}^{2}}{|\lambda_{0}\lambda_{1}|} & =\frac{\gamma_{1}^{2}+\gamma_{0}^{2}+{\left(\nu_{1}-\nu_{0}\right)}^{2}}{|\gamma_{0}\gamma_{1}|}. \end{array}$$
  Curiously, (177) implies that (V,V+1)≡(V,2V+1).
60. For location–dilation families of random variables, we saw in Item 56 how to reduce a four-parameter problem into a two-parameter problem since $\left(\lambda_{1}V+\mu_{1},\lambda_{0}V+\mu_{0}\right)\equiv \left(V,\lambda V+\mu \right)$ with the appropriate substitution. In the Cauchy case, Theorem 7 reveals that, in fact, we can go one step further and turn it into a one-parameter problem. We have two basic ways of doing this:
  (a) $\left(\lambda_{1}V+\mu_{1},\lambda_{0}V+\mu_{0}\right)\equiv \left(V,V+\mu \right)$ with $\mu^{2}=2\zeta -2$.
  (b) $\left(\lambda_{1}V+\mu_{1},\lambda_{0}V+\mu_{0}\right)\equiv \left(V,\lambda V\right)$ with either
    (178) $$\begin{array}{c} \lambda =\zeta -\sqrt{\zeta^{2}-1}<1,\;or\;\lambda =\zeta +\sqrt{\zeta^{2}-1}>1, \end{array}$$
    which are the solutions to $\zeta =\frac{\lambda^{2}+1}{2\lambda }$.

## 9. f-Divergences

This section studies the interplay of *f*-divergences and equivalent pairs of measures.

61. If P≪Q and f:[0,∞)→R is convex and right-continuous at 0, *f*-divergence is defined as
  (179) $$\begin{array}{c} D_{f}\left(P\;∥\;Q\right)=E\left[f\left(\frac{dP}{dQ}\left(Y\right)\right)\right],\;Y∼Q. \end{array}$$
62. The most important property of *f*-divergence is the data processing inequality
  (180) $$\begin{array}{c} D_{f}\left(P_{X}\;∥\;Q_{X}\right)\geq D_{f}\left(P_{Y}\;∥\;Q_{Y}\right), \end{array}$$
  where $P_{Y}$ and $Q_{Y}$ are the responses of a (random or deterministic) transformation to $P_{X}$ and $Q_{X}$, respectively. If *f* is strictly convex at 1 and $D_{f}\left(P_{X}\;∥\;Q_{X}\right)<\infty$, then $\left(P_{X},Q_{X}\right)\equiv \left(P_{Y},Q_{Y}\right)$ is necessary and sufficient for equality in (180).
63. If (P,Q)≡(Q,P), then $D_{f}\left(P\;∥\;Q\right)=D_{f^{☆}}\left(P\;∥\;Q\right)$ with the transform $f^{☆}\left(t\right)=t\;f\left(\frac{1}{t}\right)$, which satisfies $f^{☆☆}=f$.
64.
  **Theorem** **9.** *If $P_{1}\ll Q_{1}$ and $P_{2}\ll Q_{2}$, then*
(181) $$\begin{array}{c} \left(P_{1},Q_{1}\right)\equiv \left(P_{2},Q_{2}\right)\;⟺\;D_{f}\left(P_{1}\;∥\;Q_{1}\right)=D_{f}\left(P_{2}\;∥\;Q_{2}\right),\;\;\forall f, \end{array}$$
  *where ∀f stands for all convex right-continuous f:[0,∞)→R.*
  **Proof.**  As mentioned in Item 53, $\left(P_{1},Q_{1}\right)\equiv \left(P_{2},Q_{2}\right)$ is equivalent to $\frac{dP_{1}}{dQ_{1}}\left(Y_{1}\right)$ and $\frac{dP_{2}}{dQ_{2}}\left(Y_{2}\right)$ having identical distributions with $Y_{1}∼Q_{1}$ and $Y_{2}∼Q_{2}$.
  ⟹ According to (179), $D_{f}\left(P\;∥\;Q\right)$ is determined by the distribution of the random variable $\frac{dP}{dQ}\left(Y\right)$, Y∼Q.
  ⟸ For t∈R, the function $f_{t}\left(x\right)=e^{tx}$, x≥0, is convex and right-continuous at 0, and $D_{f_{t}}\left(P\;∥\;Q\right)$ is the moment generating function, evaluated at *t*, of the random variable $\frac{dP}{dQ}\left(Y\right)$, Y∼Q. Therefore, $D_{f_{t}}\left(P_{1}\;∥\;Q_{1}\right)=D_{f_{t}}\left(P_{2}\;∥\;Q_{2}\right)$ for all *t* implies that $\left(P_{1},Q_{1}\right)\equiv \left(P_{2},Q_{2}\right)$.
       □
65. Since P≪Q is not necessary in order to define (finite) $D_{f}\left(P∥Q\right)$, it is possible to enlarge the scope of Theorem 9 by defining $\left(P_{1},Q_{1}\right)\equiv \left(P_{2},Q_{2}\right)$ dropping the restriction that $P_{1}\ll Q_{1}$ and $P_{2}\ll Q_{2}$. For that purpose, let $\mu_{1}$ and $\mu_{2}$ be σ-finite measures on $\left(A_{1},F_{1}\right)$ and $\left(A_{2},F_{2}\right)$, respectively, and denote $p_{i}=\frac{dP_{i}}{d\mu_{i}}$, $q_{i}=\frac{dQ_{i}}{d\mu_{i}}$, i=1,2. Then, we say $\left(P_{1},Q_{1}\right)\equiv \left(P_{2},Q_{2}\right)$ if
  (a) when restricted to [0,1], the random variables $\frac{p_{1}\left(Y_{1}\right)}{q_{1}\left(Y_{1}\right)}$ and $\frac{p_{2}\left(Y_{2}\right)}{q_{2}\left(Y_{2}\right)}$ have identical distributions with $Y_{1}∼Q_{1}$ and $Y_{2}∼Q_{2}$;
  (b) when restricted to [0,1], the random variables $\frac{q_{1}\left(X_{1}\right)}{p_{1}\left(X_{1}\right)}$ and $\frac{q_{2}\left(X_{2}\right)}{p_{2}\left(X_{2}\right)}$ have identical distributions with $X_{1}∼P_{1}$ and $X_{2}∼P_{2}$.
  Note that those conditions imply that
  (c) $Q_{1}\left(\left\{\omega \in A_{1}:p_{1}\left(\omega \right)=q_{1}\left(\omega \right)\right\}\right)=Q_{2}\left(\left\{\omega \in A_{2}:p_{2}\left(\omega \right)=q_{2}\left(\omega \right)\right\}\right)$;
  (d) $Q_{1}\left(\left\{\omega \in A_{1}:p_{1}\left(\omega \right)=0\right\}\right)=Q_{2}\left(\left\{\omega \in A_{2}:p_{2}\left(\omega \right)=0\right\}\right)$;
  (e) $P_{1}\left(\left\{\omega \in A_{1}:q_{1}\left(\omega \right)=0\right\}\right)=P_{2}\left(\left\{\omega \in A_{2}:q_{2}\left(\omega \right)=0\right\}\right)$.
  For example, if $P_{1}⊥Q_{1}$ and $P_{2}⊥Q_{2}$, then $\left(P_{1},Q_{1}\right)\equiv \left(P_{2},Q_{2}\right)$. To show the generalized version of Theorem 9, it is convenient to use the symmetrized form
  (182) $$\begin{array}{c} D_{f}\left(P\;∥\;Q\right)=\int_{0\leq p<q}q\;f\left(\frac{p}{q}\right)\;d\mu +\int_{0\leq q<p}p\;f^{☆}\left(\frac{q}{p}\right)\;d\mu +f\left(1\right)\;Q\left[p=q\right]. \end{array}$$
66. Suppose that there is a class C of probability measures on a given measurable space with the property that there exists a convex function g:(0,∞)→R (right-continuous at 0) such that, if $\left(P_{1},Q_{1}\right)\in C^{2}$ and $\left(P_{2},Q_{2}\right)\in C^{2}$, then
  (183) $$\begin{array}{c} D_{g}\left(P_{1}\;∥\;Q_{1}\right)=D_{g}\left(P_{2}\;∥\;Q_{2}\right)\;⟺\;\left(P_{1},Q_{1}\right)\equiv \left(P_{2},Q_{2}\right). \end{array}$$
  In such case, Theorem 9 indicates that $C^{2}$ can be partitioned into equivalence classes such that, within every equivalence class, the value of $D_{f}\left(P\;∥\;Q\right)$ is constant, though naturally dependent on *f*. Throughout $C^{2}$, the value of $D_{g}\left(P\;∥\;Q\right)$ determines the value of $D_{f}\left(P\;∥\;Q\right)$, i.e., we can express $D_{f}\left(P\;∥\;Q\right)=\vartheta_{f,g}\left(D_{g}\left(P\;∥\;Q\right)\right)$, where $\vartheta_{f,g}$ is a non-decreasing function. Consider the following examples:
  (a) Let C be the class of real-valued Gaussian probability measures with given variance $\sigma^{2}>0$. Then,
    (184) $$\begin{array}{c} D\left(N\;\left(\mu_{1},\sigma^{2}\right)\;∥\;N\;\left(\mu_{2},\sigma^{2}\right)\right)=\frac{{\left(\mu_{1}-\mu_{2}\right)}^{2}}{\sigma^{2}}\log e. \end{array}$$
    Since Theorem 8 implies that $\left(N\;\left(\mu_{1},\sigma^{2}\right),N\;\left(\mu_{2},\sigma^{2}\right)\right)\equiv \left(N\;\left(\mu_{3},\sigma^{2}\right),N\;\left(\mu_{4},\sigma^{2}\right)\right)$ as long as ${\left(\mu_{1}-\mu_{2}\right)}^{2}={\left(\mu_{3}-\mu_{4}\right)}^{2}$, (184) indicates that (183) is satisfied with g(t) given by the right-continuous extension of tlogt. Therefore, we can conclude that, regardless of *f*, $D_{f}\left(N\;\left(\mu_{1},\sigma^{2}\right)\;∥\;N\;\left(\mu_{2},\sigma^{2}\right)\right)$ depends on $\left(\mu_{1},\mu_{2},\sigma^{2}\right)$ only through ${\left(\mu_{1}-\mu_{2}\right)}^{2}/\sigma^{2}$.
  (b) Let C be the collection of all Cauchy random variables. Theorem 7 reveals that (183) is also satisfied if $g\left(x\right)=x^{2}$ because, if X∼P and Y∼Q, then
    (185) $$\begin{array}{c} E\left[\frac{dP}{dQ}\left(X\right)\right]=E\left[{\left(\frac{dP}{dQ}\left(Y\right)\right)}^{2}\right]. \end{array}$$
67. An immediate consequence of Theorems 7 and 9 is that, for any valid *f*, the *f*-divergence between Cauchy densities is symmetric,
  (186) $$\begin{array}{c} D_{f}\left(\lambda_{1}V+\mu_{1}\;∥\;\lambda_{0}V+\mu_{0}\right)=D_{f}\left(\lambda_{0}V+\mu_{0}\;∥\;\lambda_{1}V+\mu_{1}\right). \end{array}$$
  This property does not generalize to the multivariate case. While, in view of Theorem 8,
  (187) $$\begin{array}{c} \left(\Lambda^{\frac{1}{2}}V^{n}+\mu_{1},\Lambda^{\frac{1}{2}}V^{n}+\mu_{2}\right)\equiv \left(\Lambda^{\frac{1}{2}}V^{n}+\mu_{2},\Lambda^{\frac{1}{2}}V^{n}+\mu_{1}\right), \end{array}$$
  in general, $\left(\Lambda^{\frac{1}{2}}V^{n},V^{n}\right)≢\left(V^{n},\Lambda^{\frac{1}{2}}V^{n}\right)$ since the corresponding relative entropies do not coincide as shown in [8].
68. It follows from Item 66 and Theorem 7 that any *f*-divergence between Cauchy probability measures $D_{f}\left(\lambda_{1}V+\mu_{1}\;∥\;\lambda_{0}V+\mu_{0}\right)$ is a monotonically increasing function of $\zeta (\lambda_{1},\mu_{1},\lambda_{0},\mu_{0})$ given by (149). The following result shows how to obtain that function from *f*.
  **Theorem** **10.** *With $f_{Z}$ given in (153),*
(188) $$\begin{array}{cc} D_{f}\left(\lambda_{1}V+\mu_{1}\;∥\;\lambda_{0}V+\mu_{0}\right)\; & =\int_{\zeta -\sqrt{\zeta^{2}-1}}^{\zeta +\sqrt{\zeta^{2}-1}}f\left(\frac{1}{z}\right)\;f_{Z}\left(z\right)\;dz \end{array}$$
(189) $$\begin{array}{cc} \; & =E\left[f\left({\left(\zeta +\sqrt{\zeta^{2}-1}\;\cos\Theta \right)}^{-1}\right)\right] \end{array}$$
(190) $$\begin{array}{cc} \; & =\int_{\zeta -\sqrt{\zeta^{2}-1}}^{\zeta +\sqrt{\zeta^{2}-1}}\frac{1}{z}\;f\left(z\right)\;f_{Z}\left(z\right)\;dz. \end{array}$$
  *where Θ is uniformly distributed on [0,π] in (189).*
  **Proof.**  In view of (179) and the definition of *Z* in Theorem 7,
  (191) $$\begin{array}{c} D_{f}\left(\lambda_{1}V+\mu_{1}\;∥\;\lambda_{0}V+\mu_{0}\right)=E\left[f\left(\frac{1}{Z}\right)\right], \end{array}$$
  thereby justifying (188) and (189) since we saw in Theorem 7 that *Z* has the distribution of $\zeta +\sqrt{\zeta^{2}-1}\;\cos\Theta$ with Θ uniformly distributed on [0,π]. Item 52 results in (190). Alternatively, we can rely on Item 63 and substitute *f* by $f^{☆}$ on the right side of (188).    □
69. Suppose now that we have two sequences of Cauchy measures with respective parameters $\left(\lambda_{1}^{\left(n\right)},\mu_{1}^{\left(n\right)}\right)$ and $\left(\lambda_{0}^{\left(n\right)},\mu_{0}^{\left(n\right)}\right)$ such that $\zeta (\lambda_{1}^{\left(n\right)},\mu_{1}^{\left(n\right)},\lambda_{0}^{\left(n\right)},\mu_{0}^{\left(n\right)})\to 1$. Then, Theorem 10 indicates that
  (192) $$\begin{array}{c} D_{f}\left(\lambda_{1}^{\left(n\right)}V+\mu_{1}^{\left(n\right)}\;∥\;\lambda_{0}^{\left(n\right)}V+\mu_{0}^{\left(n\right)}\right)\to f\left(1\right). \end{array}$$
  The most common *f*-divergences are such that f(1)=0 since in that case $D_{f}\left(P∥Q\right)\geq 0$. In addition, adding the function αt−α to f(t) does not change the value of $D_{f}\left(P∥Q\right)$ and with appropriately chosen α, we can turn f(t) into canonical form in which not only f(1)=0 but f(t)≥0. In the special case in which the second measure is fixed, Theorem 9 in [25] shows that, if $\mathrm{ess}\sup\frac{dP_{n}}{dQ}\left(Y\right)\to 1$ with Y∼Q, then
  (193) $$\begin{array}{c} \lim_{n\to \infty }\frac{D_{f}\left(P_{n}∥Q\right)}{D_{g}\left(P_{n}∥Q\right)}=\lim_{t\to 1}\frac{f(t)}{g(t)}, \end{array}$$
  provided the limit on the right side exists; otherwise, the left side lies between the left and right limits at 1. In the Cauchy case, we can allow the second probability to depend on *n* and sharpen that result by means of Theorem 10. In particular, it can be shown that
  (194) $$\begin{array}{c} \lim_{n\to \infty }\frac{D_{f}\left(\lambda_{1}^{\left(n\right)}V+\mu_{1}^{\left(n\right)}\;∥\;\lambda_{0}^{\left(n\right)}V+\mu_{0}^{\left(n\right)}\right)}{D_{g}\left(\lambda_{1}^{\left(n\right)}V+\mu_{1}^{\left(n\right)}\;∥\;\lambda_{0}^{\left(n\right)}V+\mu_{0}^{\left(n\right)}\right)}=\frac{\dot{f}\left(0^{-}\right)+\dot{f}\left(0^{+}\right)}{\dot{g}\left(0^{-}\right)+\dot{g}\left(0^{+}\right)} \end{array}$$
  provided the right side is not $\frac{0}{0}$.

## 10. $\chi^{2}$-Divergence

70. With either $f\left(x\right)={\left(x-1\right)}^{2}$ or $f\left(x\right)=x^{2}-1$, *f*-divergence is the $\chi^{2}$-*divergence*,
  (195) $$\begin{array}{cc} \chi^{2}\left(P\;∥\;Q\right) & =E\left[\frac{dP}{dQ}\left(X\right)\right]-1,\;\;X∼P. \end{array}$$
71. If *P* and *Q* are Cauchy distributions, then (149), (151) and (195) result in
  (196) $$\begin{array}{cc} \chi^{2}\left(\lambda_{1}V+\mu_{1}\;∥\;\lambda_{0}V+\mu_{0}\right) & =\zeta (\lambda_{1},\mu_{1},\lambda_{0},\mu_{0})-1 \end{array}$$
  (197) $$\begin{array}{cc} \; & =\frac{\left(\right|\lambda_{0}\left|-\right|\lambda_{1}{\left|\right)}^{2}+{\left(\mu_{1}-\mu_{0}\right)}^{2}}{2\;|\lambda_{0}\;\lambda_{1}|}, \end{array}$$
  a formula obtained in Appendix D of [26] using complex analysis and the Cauchy integral formula. In addition, invoking complex analysis and the maximal group invariant results in [27,28], ref. [26] shows that any *f*-divergence between Cauchy distributions can be expressed as a function of their $\chi^{2}$ divergence, although [26] left open how to obtain that function, which is given by Theorem 10 substituting $\zeta =1+\chi^{2}$.

## 11. Relative Entropy

72. The relative entropy between Cauchy distributions is given by
  (198) $$\begin{array}{c} D\left(\lambda_{1}V+\mu_{1}\;∥\;\lambda_{0}V+\mu_{0}\right)=\log\left(\frac{\left(\right|\lambda_{0}\left|+\right|\lambda_{1}{\left|\right)}^{2}+{\left(\mu_{1}-\mu_{0}\right)}^{2}}{4\;|\lambda_{0}\;\lambda_{1}|}\right), \end{array}$$
  where $\lambda_{1}\lambda_{0}\neq 0$. The special case $\lambda_{1}=\lambda_{0}$ of (198) was found in Example 4 of [29]. The next four items give different simple justifications for (198). An alternative proof was recently given in Appendix C of [26] using complex analysis holomorphisms and the Cauchy integral formula. Yet another, much more involved, proof is reported in [30]. See also Remark 19 in [26] for another route invoking the Lévy–Khintchine formula and the Frullani integral.
73. Since for absolutely continuous random variables $D\left(X\;∥\;Y\right)=-h\left(X\right)-E[\log f_{Y}\left(X\right)]$,
  (199) $$\begin{array}{cc} D\left(V\;∥\;\lambda \;V+\mu \right)\; & =-h\left(V\right)+\log\frac{\pi }{\left|\lambda \right|}+E\left[\log\left(\lambda^{2}+{\left(V-\mu \right)}^{2}\right)\right] \end{array}$$
  (200) $$\begin{array}{cc} \; & =-\log\left(4|\lambda |\right)+\log\left({\left(1+|\lambda |\right)}^{2}+\mu^{2}\right), \end{array}$$
  where (200) follows from (12) and (A4) with $\alpha^{2}=\lambda^{2}+\mu^{2}$ and $\cos\beta =\frac{\mu }{\left|\alpha \right|}.$
  Now, substituting $\lambda =\frac{\lambda_{0}}{\lambda_{1}}$ and $\mu =\frac{\mu_{0}-\mu_{1}}{\lambda_{1}}$, we obtain (198) since, according to Item 56, $\left(V,\lambda \;V+\mu \right)\equiv \left(\lambda_{1}V+\mu_{1},\lambda_{0}V+\mu_{0}\right)$.
74. From the formula found in Example 4 of [29] and the fact that, according to (197), $\chi^{2}=\frac{\mu^{2}}{2\lambda^{2}}$ when $\lambda_{1}=\lambda_{0}=\lambda$, we obtain
  (201) $$\begin{array}{c} D\left(\lambda V+\mu \;∥\;\lambda V\right)=\log\left(1+\frac{\mu^{2}}{4\lambda^{2}}\right)=\log\left(1+\frac{1}{2}\chi^{2}\right). \end{array}$$
  Moreover, as argued in Item 60, (201) is also valid for the relative entropy between Cauchy distributions with $\lambda_{1}\neq \lambda_{0}$ as long as $\chi^{2}$ is given in (197). Indeed, we can verify that the right side of (201) becomes (198) with said substitution.
75. By the definition of relative entropy, and Theorem 7,
  (202) $$\begin{array}{cc} D(\lambda_{1}V+\mu_{1}\;∥\;\lambda_{0}V+\mu_{0}) & =E\left[\log Z\right] \end{array}$$
  (203) $$\begin{array}{cc} \; & =\frac{1}{2\pi }\int_{0}^{2\pi }\log\left(\zeta +\sqrt{\zeta^{2}-1}\;\cos\theta \right)\;d\theta \end{array}$$
  (204) $$\begin{array}{cc} \; & =\log\left(\frac{1+\zeta }{2}\right), \end{array}$$
  where (204) follows from (A14). Then, (198) results by plugging into (204) the value of ζ in (149).
76. Evaluating (190) with f(t)=tlogt results in (202).
77. If *V* is standard Cauchy, independent of Cauchy $V_{1}$ and $V_{0}$, then (198) results in
  (205) $$\begin{array}{c} D\left(\lambda V+\epsilon \;V_{1}\;∥\;\lambda V+\epsilon \;V_{0}\right)=\frac{\epsilon^{2}}{4\lambda^{2}}\left({\left(\lambda_{1}-\lambda_{0}\right)}^{2}+{\left(\mu_{1}-\mu_{0}\right)}^{2}\right)\log e+o\left(\epsilon^{2}\right), \end{array}$$
  where $V_{1}=\lambda_{1}V^{\prime }+\lambda_{1}$ and $V_{0}=\lambda_{1}V^{\prime }+\lambda_{1}$, and $V^{\prime }$ is an independent (or exact) copy of *V*. In contrast, the corresponding result in the Gaussian case in which *X*, $X_{1}$, $X_{0}$ are independent Gaussian with means $\mu ,\mu_{1},\mu_{0}$ and variances $\sigma^{2},\sigma_{1}^{2},\sigma_{0}^{2}$, respectively, is
  (206) $$\begin{array}{c} D\left(X+\epsilon \;X_{1}\;∥\;X+\epsilon \;X_{0}\right)=\frac{\epsilon^{2}}{2\sigma^{2}}{\left(\mu_{1}-\mu_{0}\right)}^{2}\log e+o\left(\epsilon^{2}\right). \end{array}$$
  In fact, it is shown in Lemma 1 of [31] that (206) holds even if $X_{1}$ and $X_{0}$ are not Gaussian but have finite variances. It is likely that (205) holds even if $V_{1}$ and $V_{0}$ are not Cauchy, but have finite strengths.
78. An important information theoretic result due to Csiszár [32] is that if $Q_{1}\ll Q_{2}$ and *P* is such that
  (207) $$\begin{array}{cc} E\left[{ı}_{Q_{1}∥Q_{2}}\left(X\right)\right] & =D(Q_{1}\;∥\;Q_{2}),\;X∼P, \end{array}$$
  then the following *Pythagorean identity* holds
  (208) $$\begin{array}{cc} D(P\;∥\;Q_{2}) & =D\left(P\;∥\;Q_{1}\right)+D\left(Q_{1}\;∥\;Q_{2}\right). \end{array}$$
  Among other applications, this result leads to elegant proofs of minimum relative entropy results. For example, the closest Gaussian to a given *P* with a finite second moment has the same first and second moments as *P*. If we let $Q_{1}$ and $Q_{2}$ be centered Cauchy with strengths $\lambda_{1}$ and $\lambda_{2}$, respectively, then the orthogonality condition (207) becomes, with the aid of (148) and (198),
  (209) $$\begin{array}{c} V_{X^{2}}\left(\lambda_{2}^{-1}\right)-V_{X^{2}}\left(\lambda_{1}^{-1}\right)=2\;\log_{e}\left(1+\frac{\lambda_{1}}{\lambda_{2}}\right)-2\log_{e}2. \end{array}$$
  If, in addition, *P* is centered Cauchy, we can use (28) to verify that (209) holds only in the trivial cases in which either $\lambda_{1}=\lambda_{2}$ or $P=Q_{1}$. For non-Cauchy *P*, (208) may indeed be satisfied with $\lambda_{1}\neq \lambda_{2}$. For example, using (30), if $X=V_{2,2}$, then (209), and therefore (208), holds with $\left(\lambda_{1},\lambda_{2}\right)=\left(2,0.35459\ldots \right)$.
79. Mutually absolutely continuous random variables may be such that
  (210) $$\begin{array}{c} D(X\;∥\;Z)<\infty =D(Z\;∥\;X). \end{array}$$
  An easy example is that of Gaussian *X* and Cauchy *Z*, or, if we let *X* be Cauchy, (210) holds with *Z* having the very heavy-tailed density function in (62).
80. While relative entropy is lower semi-continuous, it is not continuous. For example, using the Cauchy distribution, we can show that relative entropy is not stable against small contamination of a Gaussian random variable: if *X* is Gaussian independent of *V*, then no matter how small λ≠0,
  (211) $$\begin{array}{c} D(\lambda |V|+X\;∥\;-\lambda |V|+X)=\infty . \end{array}$$

## 12. Total Variation Distance

81. With f(x)=|x−1|, *f*-divergence becomes the *total variation distance* (with range [0,2]). Moreover, we have the following representation:
  **Theorem** **11.** *If P≪≫Q and (P,Q)≡(Q,P), then*
  (212) $$\begin{array}{c} \frac{1}{2}\left|P-Q\right|=2\;P\left[Z>1\right]-P\left[Z\neq 1\right], \end{array}$$
  *with $Z=\frac{dP}{dQ}\left(X\right)$, X∼P.*
  **Proof.**  (213) $$\begin{array}{cc} \frac{1}{2}\left|P-Q\right| & =\max_{A\in F}\left\{P(A)-Q(A)\right\} \end{array}$$
  (214) $$\begin{array}{cc} \; & =P\left(\omega :\frac{dP}{dQ}\left(\omega \right)>1\right)-Q\left(\omega :\frac{dP}{dQ}\left(\omega \right)>1\right) \end{array}$$
  (215) $$\begin{array}{cc} \; & =P\left(\omega :\frac{dP}{dQ}\left(\omega \right)>1\right)-P\left(\omega :\frac{dQ}{dP}\left(\omega \right)>1\right) \end{array}$$
  (216) $$\begin{array}{cc} \; & =P[Z>1]-P[Z<1] \end{array}$$
  where (215) and (216) follow from (P,Q)≡(Q,P) and P≪≫Q, respectively.    □
82. Example 15 of [33] shows that the total variation distance between centered Cauchy distributions is
  (217) $$\begin{array}{cc} \left|P_{\lambda_{1}V}-P_{\lambda_{0}V}\right| & =\frac{4}{\pi }\arctan\left(\frac{\left|\right|\lambda_{1}\left|-\right|\lambda_{0}\left|\right|}{2\sqrt{|\lambda_{0}\lambda_{1}|}}\right) \end{array}$$
  (218) $$\begin{array}{cc} \; & =\frac{4}{\pi }\arctan\left(\sqrt{\frac{1}{2}\;\chi^{2}\left(P_{\lambda_{1}V}∥P_{\lambda_{0}V}\right)}\right) \end{array}$$
  in view of (197). Since any *f*-divergence between Cauchy distributions depends on the parameters only through the corresponding $\chi^{2}$-divergence, (217)–(218) imply the general formula
  (219) $$\begin{array}{c} \left|P_{\lambda_{1}V+\mu_{1}}-P_{\lambda_{0}V+\mu_{0}}\right|=\frac{4}{\pi }\arctan\left(\sqrt{\frac{1}{2}\;\chi^{2}\left(P_{\lambda_{1}V+\mu_{1}}∥P_{\lambda_{0}V+\mu_{0}}\right)}\right). \end{array}$$
  Alternatively, applying Theorem 11 to the case of Cauchy random variables, note that, in this case, *Z* is an absolutely continuous random variable with density function (153). Therefore, P[Z≠1] = 1, and
  (220) $$\begin{array}{cc} P[Z>1] & =\frac{1}{\pi }\int_{1}^{\zeta +\sqrt{\zeta^{2}-1}}\frac{1}{\sqrt{2z\zeta -z^{2}-1}}\;dz \end{array}$$
  (221) $$\begin{array}{cc} \; & =\frac{1}{2}+\frac{1}{\pi }\arctan\sqrt{\frac{1}{2}\chi^{2}}, \end{array}$$
  where (221) follows from (154) and the identity $\arcsin\frac{\sqrt{\delta }}{\sqrt{1+\delta }}=\arctan\sqrt{\delta }$ specialized to $\delta =\frac{1}{2}\chi^{2}=\frac{1}{2}\left(\zeta -1\right)$. Though more laborious (see [26]), (219) can also be verified by direct integration.

## 13. Hellinger Divergence

83. The Hellinger divergence, $H_{\alpha }\left(P\;∥\;Q\right)$ of order α∈(0,1)∪(1,∞), is the $f_{\alpha }$-divergence with
  (222) $$\begin{array}{c} f_{\alpha }\left(t\right)=\frac{t^{\alpha }-1}{\alpha -1}. \end{array}$$
  Notable special cases are
  (223) $$\begin{array}{cc} H_{2}\left(P\;∥\;Q\right) & =\chi^{2}\left(P\;∥\;Q\right), \end{array}$$
  (224) $$\begin{array}{cc} \lim_{\alpha ↓1}H_{\alpha }\left(P\;∥\;Q\right) & =D(P\;∥\;Q), \end{array}$$
  (225) $$\begin{array}{cc} H_{\frac{1}{2}}\left(P\;∥\;Q\right) & =2\;H^{2}\left(P\;∥\;Q\right), \end{array}$$
  where $H^{2}\left(P\;∥\;Q\right)$ is known as the squared Hellinger distance.
84. For Cauchy random variables, Theorem 10 yields
  (226) $$\begin{array}{cc} H_{\alpha }\left(\lambda_{1}V+\mu_{1}\;∥\;\lambda_{0}V+\mu_{0}\right) & =\frac{1}{\alpha -1}\left(E[Z^{-\alpha }]-1\right) \end{array}$$
  (227) $$\begin{array}{cc} \; & =\frac{P_{\alpha -1}\left(\zeta \right)-1}{\alpha -1}, \end{array}$$
  where ζ is as given in (149), and we have used (A15) and $P_{\alpha }\left(\cdot \right)$ denotes the Legendre function of the first kind, which satisfies $P_{-\alpha }=P_{\alpha -1}$ (see 8.2.1. in [34]).

## 14. Rényi Divergence

85. For absolutely continuous probability measures *P* and *Q*, with corresponding probability density functions *p* and *q*, the *Rényi divergence of order*α∈[0,1)∪(1,∞) is [35]
  (228) $$\begin{array}{cc} D_{\alpha }\left(P\;∥\;Q\right)\; & =\frac{1}{\alpha -1}\log\left(\int_{-\infty }^{\infty }p^{\alpha }\left(t\right)\;q^{1-\alpha }\left(t\right)\;dt\right). \end{array}$$
  Note that, if $\left(P_{1},Q_{1}\right)\equiv \left(P_{2},Q_{2}\right)$, then $D_{\alpha }\left(P_{1}\;∥\;Q_{1}\right)=D_{\alpha }\left(P_{2}\;∥\;Q_{2}\right)$. Moreover, although Rényi divergence of order α is not an *f*-divergence, it is in one-to-one correspondence with the Hellinger divergence of order α:
  (229) $$\begin{array}{c} D_{\alpha }\left(P\;∥\;Q\right)=\frac{1}{\alpha -1}\log\left(1+\left(\alpha -1\right)\;H_{\alpha }\left(P\;∥\;Q\right)\right). \end{array}$$
86. An extensive table of order-α Rényi divergences for various continuous random variables can be found in [36]. An addition to that list for Cauchy random variables can be obtained plugging (227) into (229):
  (230) $$\begin{array}{cc} D_{\alpha }\left(\lambda_{1}V+\mu_{1}\;∥\;\lambda_{0}V+\mu_{0}\right) & =\frac{\log P_{\alpha -1}\left(\zeta \right)}{\alpha -1} \end{array}$$
  (231) $$\begin{array}{cc} \; & =\frac{1}{\alpha -1}\log P_{\alpha -1}\left(\frac{\lambda_{1}^{2}+\lambda_{0}^{2}+{\left(\mu_{1}-\mu_{0}\right)}^{2}}{2|\lambda_{0}\lambda_{1}|}\right), \end{array}$$
  for α∈(0,1)∪(1,∞).
87. Suppose that λ∈(0,1). Then, (A16) yields
  (232) $$\begin{array}{c} D_{\frac{1}{2}}\left(V\;∥\;\lambda V\right)=-2\log\left(\frac{2\sqrt{\lambda }}{\pi }\;K\left(\sqrt{1-\lambda^{2}}\right)\right), \end{array}$$
  where K(·) stands for the complete elliptical integral of the first kind in (A18). As indicated in Item 60, to obtain $D_{\frac{1}{2}}\left(\lambda_{1}V+\mu_{1}\;∥\;\lambda_{0}V+\mu_{0}\right)$, we just need to substitute λ by $\zeta -\sqrt{\zeta^{2}-1}$ in (232), with ζ given by (149).
88. Notice that, specializing (86) to $\left(\alpha ,\mu_{0},\mu_{1},\lambda_{0},\lambda_{1}\right)=\left(\frac{1}{2},0,0,\lambda ,1\right)$, (232) results in the identity
  (233) $$\begin{array}{c} P_{-\frac{1}{2}}\left(\frac{1}{2\lambda }+\frac{\lambda }{2}\right)=\frac{2\sqrt{\lambda }}{\pi }\;K\left(\sqrt{1-\lambda^{2}}\right),\;\lambda \in \left(0,1\right). \end{array}$$
  Writing the complete elliptical integral of the first kind and the Legendre function of the first kind as special cases of the Gauss hypergeometric function, González [37] noticed the simpler identity (see also 8.13.8 in [34])
  (234) $$\begin{array}{c} P_{-\frac{1}{2}}\left(\lambda \right)=\frac{2}{\pi }\;K\left(\sqrt{\frac{1-\lambda }{2}}\right),\;\lambda \in \left(0,1\right). \end{array}$$
  We can view (233) and (234) as complementary of each other since they constrain the argument of the Legendre function to belong to (1,∞) and (0,1), respectively.
89. Since $P_{1}\left(z\right)=z$, particularizing (230), we obtain
  (235) $$\begin{array}{cc} D_{2}\left(\lambda_{1}V+\mu_{1}\;∥\;\lambda_{0}V+\mu_{0}\right)=\log\zeta \; & =\log\left(\frac{\lambda_{1}^{2}+\lambda_{0}^{2}+{\left(\mu_{1}-\mu_{0}\right)}^{2}}{2|\lambda_{0}\lambda_{1}|}\right). \end{array}$$
90. Since $P_{2}\left(z\right)=\frac{1}{2}\left(3z^{2}-1\right)$, for Cauchy random variables, we obtain
  (236) $$\begin{array}{c} D_{3}\left(P\;∥\;Q\right)=\frac{1}{2}\log\left(1+3\;\chi^{2}\left(P\;∥\;Q\right)+\frac{3}{2}\;\chi^{4}\left(P\;∥\;Q\right)\right). \end{array}$$
91. For Cauchy random variables, the Rényi divergence for integer order 4 or higher can be obtained through (235), (236) and the recursion (dropping (P∥Q) for typographical convenience)
  (237) $$\begin{array}{c} \left(n+1\right)\exp\left(\left(n+1\right)D_{n+2}\right)=\left(2n+1\right)\;\zeta \;\exp\left(nD_{n+1}\right)-n\exp\left(\left(n-1\right)D_{n}\right), \end{array}$$
  which follows from (230) and the recursion of the Legendre polynomials
  (238) $$\begin{array}{c} \left(n+1\right)\;P_{n+1}\left(z\right)=\left(2n+1\right)\;z\;P_{n}\left(z\right)-n\;P_{n-1}\left(z\right), \end{array}$$
  which, in fact, also holds for non-integer *n* (see 8.5.3 in [34]).
92. The Chernoff information
  (239) $$\begin{array}{c} C\left(P\;∥\;Q\right)=\underset{\lambda \in (0,1)}{\sup}\left(1-\lambda \right)D_{\lambda }\left(P\;∥\;Q\right) \end{array}$$
  satisfies C(P∥Q)=C(Q∥P) regardless of (P,Q). If, as in the case of Cauchy measures, (P,Q)≡(Q,P), then Chernoff information is equal to the Bhattacharyya distance:
  (240) $$\begin{array}{c} C\left(P\;∥\;Q\right)=\frac{1}{2}D_{\frac{1}{2}}\left(P\;∥\;Q\right)=\log\frac{1}{\int_{-\infty }^{\infty }\sqrt{p(t)\;q(t)}\;dt}=-\log\left(1-H^{2}\left(P∥Q\right)\right), \end{array}$$
  where $H^{2}\left(P∥Q\right)$ is the squared Hellinger distance, which is the *f*-divergence with $f\left(t\right)=\frac{1}{2}{\left(1-\sqrt{t}\right)}^{2}$. Together with Item 87, (240) gives the Chernoff information for Cauchy distributions. While it involves the complete elliptical integral function, its simplicity should be contrasted with the formidable expression for Gaussian distributions, recently derived in [38]. The reason (240) holds is that the supremum in (239) is achieved at $\lambda^{*}=\frac{1}{2}$. To see this, note that
  (241) $$\begin{array}{cc} f\left(\lambda \right)=\left(1-\lambda \right)D_{\lambda }\left(P\;∥\;Q\right) & =\lambda \;D_{1-\lambda }\left(Q\;∥\;P\right) \end{array}$$
  (242) $$\begin{array}{cc} \; & =\lambda \;D_{1-\lambda }\left(P\;∥\;Q\right) \end{array}$$
  (243) $$\begin{array}{cc} \; & =f(1-\lambda ), \end{array}$$
  where (241) reflects the skew-symmetry of Rényi divergence, and (242) holds because (P,Q)≡(Q,P). Since f(λ):λ∈[0,1] is concave and its own mirror image, it is maximized at $\lambda^{*}=\frac{1}{2}$.

## 15. Fisher’s Information

93. The score function of the standard Cauchy density (1) is
  (244) $$\begin{array}{c} \rho_{V}\left(x\right)=\nabla \log_{e}f_{V}\left(x\right)=-\nabla \log_{e}\left(1+x^{2}\right)=-\frac{2\;x}{1+x^{2}}. \end{array}$$
  Then, $\rho_{V}\left(V\right)$ is a zero-mean random variable with second moment equal to Fisher’s information
  (245) $$\begin{array}{c} J\left(V\right)=E\left[\rho_{V}^{2}\left(V\right)\right]=\frac{1}{\pi }\int_{-\infty }^{\infty }\frac{4\;t^{2}}{{\left(1+t^{2}\right)}^{3}}\;dt=\frac{1}{2}, \end{array}$$
  where we have used (A11). Since Fisher’s information is invariant to location and scales as $J\left(X\right)=\alpha^{2}\;J\left(\alpha X\right)$, we obtain
  (246) $$\begin{array}{c} J\left(\lambda V+\mu \right)=\frac{1}{2\;\lambda^{2}}. \end{array}$$
  Together with (117), the product of entropy power and Fisher information is $\frac{4\pi }{e}$, thereby abiding by Stam’s inequality [4], 1≤N(X)J(X).
94. Introduced in [39], Fisher’s information of a density function (245) quantifies its similarity with a slightly shifted version of itself. A more general notion is the Fisher information matrix of a random transformation $P_{Y|X}:R^{k}\to Y$ satisfying the regularity condition
  (247) $$\begin{array}{c} D\left(P_{Y|X=\alpha }\;∥\;P_{Y|X=\theta }\right)=o(∥\alpha -\theta ∥). \end{array}$$
  Then, the Fisher information matrix of $P_{Y|X}$ at θ has coefficients
  (248) $$\begin{array}{cc} J_{ij}\left(\theta ,P_{Y|X}\right) & =E\left[\frac{\partial }{\partial \alpha_{i}}{ı}_{P_{Y|X=\alpha }∥P_{Y|X=\theta }}\left(Y_{\theta }\right)\frac{\partial }{\partial \alpha_{j}}{ı}_{P_{Y|X=\alpha }∥P_{Y|X=\theta }}\left(Y_{\theta }\right)\right]{|}_{\alpha \leftarrow \theta }, \end{array}$$
  and satisfies (with relative entropy in nats)
  (249) $$\begin{array}{c} D\left(P_{Y|X=\alpha }\;∥\;P_{Y|X=\theta }\right)=\frac{1}{2}{\left(\alpha -\theta \right)}^{⊤}J\left(\theta ,P_{Y|X}\right)\left(\alpha -\theta \right){+o(∥\alpha -\theta ∥}^{2}). \end{array}$$
  For the Cauchy family, the parametrization vector has two components, location and strength, namely, $\theta^{⊤}=\left(\mu ,\lambda \right)$. The regularity condition (247) is satisfied in view of (205), and we can use the closed-form expression in (205) to obtain
  (250) $$\begin{array}{cc} J_{11}\left(\theta ,P_{Y|X}\right)=J_{22}\left(\theta ,P_{Y|X}\right) & =\frac{1}{2\;\lambda^{2}}, \end{array}$$
  (251) $$\begin{array}{cc} J_{12}\left(\theta ,P_{Y|X}\right)=J_{21}\left(\theta ,P_{Y|X}\right) & =0. \end{array}$$
95. The relative Fisher information is defined as
  (252) $$\begin{array}{c} J\left(P\;∥\;Q\right)=E\left[{\left(\nabla {ı}_{P∥Q}\left(X\right)\right)}^{2}\right],\;X∼P. \end{array}$$
  Although the purpose of this definition is to avoid some of the pitfalls of the classical definition of Fisher’s information, not only do equivalent pairs fail to have the same relative Fisher information but, unlike relative entropy or *f*-divergence, relative Fisher information is not transparent to injective transformations. For example, $J\left(X∥Y\right)=\lambda^{2}J\left(\lambda X∥\;\lambda Y\right)$. Centered Cauchy random variables illustrate this fact since
  (253) $$\begin{array}{c} J\left(V\;∥\;\lambda V\right)=\frac{\left(4+\lambda \right){\left(\lambda -1\right)}^{2}}{2\lambda {\left(1+\lambda \right)}^{2}}\;\mathrm{and}\;J\left(\lambda V\;∥\;V\right)=\frac{\left(4\lambda +1\right){\left(\lambda -1\right)}^{2}}{2\lambda^{2}{\left(1+\lambda \right)}^{2}}. \end{array}$$
96. de Bruijn’s identity [4] states that, if $N∼N\;\left(0,1\right)$ is independent of *X*, then, in nats,
  (254) $$\begin{array}{c} \frac{d}{dt}h\left(X+\sqrt{t}\;N\right)=\frac{1}{2}\;J\left(X+\sqrt{t}\;N\right),\;\;t>0. \end{array}$$
  As well as serving as the key component in the original proofs of the entropy power inequality, the differential equation in (254) provides a concrete link between Shannon theory and its prehistory. As we show in Theorem 12, it turns out that there is a Cauchy counterpart of de Bruijn’s identity (254). Before stating the result, we introduce the following notation for a parametrized random variable $Y_{t}$ (to be specified later):
  (255) $$\begin{array}{cc} \nabla \log_{e}f_{Y_{t}}\left(y\right) & =\frac{\partial }{\partial y}\log_{e}f_{Y_{t}}\left(y\right)=f_{Y_{t}}^{-1}\left(y\right)\frac{\partial }{\partial y}f_{Y_{t}}\left(y\right), \end{array}$$
  (256) $$\begin{array}{cc} \nabla_{2}\;\log_{e}f_{Y_{t}}\left(y\right) & =\frac{\partial }{\partial t}\log_{e}f_{Y_{t}}\left(y\right)=f_{Y_{t}}^{-1}\left(y\right)\frac{\partial }{\partial t}f_{Y_{t}}\left(y\right), \end{array}$$
  (257) $$\begin{array}{cc} J(Y_{t}) & =E\left[{\left(\nabla \log_{e}f_{Y_{t}}\left(Y_{t}\right)\right)}^{2}\right], \end{array}$$
  (258) $$\begin{array}{cc} K(Y_{t}) & =E\left[{\left(\nabla_{2}\;\log_{e}f_{Y_{t}}\left(Y_{t}\right)\right)}^{2}\right], \end{array}$$
  i.e., $J(Y_{t})$ and $K(Y_{t})$ are the Fisher information with respect to location and with respect to dilation, respectively (corresponding to the coefficients $J_{11}$ and $J_{22}$ of the Fisher information matrix when $\theta^{⊤}=\left(\mu ,\lambda \right)$ as in Item 94. The key to (254) is that $Y_{t}=X+\sqrt{t}\;N$, $N∼N\;\left(0,1\right)$ satisfies the partial differential equation
  (259) $$\begin{array}{c} \frac{\partial^{2}}{\partial y^{2}}f_{Y_{t}}\left(y\right)=\frac{\partial }{\partial t}f_{Y_{t}}\left(y\right). \end{array}$$
  **Theorem** **12.** *Suppose that X is independent of standard Cauchy V. Then, in nats,*
(260) $$\begin{array}{c} \frac{d^{2}}{dt^{2}}h\left(X+t\;V\right)=-J\left(X+t\;V\right)-K\left(X+t\;V\right),\;t>0. \end{array}$$
  **Proof.**  Equation (259) does not hold in the current case in which $Y_{t}=X+t\;V$, and
  (261) $$\begin{array}{c} f_{Y_{t}}\left(y\right)=\frac{t}{\pi }\;E\left[\frac{1}{t^{2}+{\left(X-y\right)}^{2}}\right]. \end{array}$$
  However, some algebra (the differentiation/integration swaps can be justified invoking the bounded convergence theorem) indicates that the convolution with the Cauchy density satisfies the Laplace partial differential equation
  (262) $$\begin{array}{c} \frac{\partial^{2}}{\partial y^{2}}f_{Y_{t}}\left(y\right)=-\frac{\partial^{2}}{\partial t^{2}}f_{Y_{t}}\left(y\right)=\frac{2t}{\pi }\;E\left[\frac{3{\left(X-y\right)}^{2}-t^{2}}{{\left(t^{2}+{\left(X-y\right)}^{2}\right)}^{3}}\right]. \end{array}$$
  The derivative of the differential entropy of $Y_{t}$ is, in nats,
  (263) $$\begin{array}{cc} \frac{d}{dt}h\left(Y_{t}\right) & =-\int_{-\infty }^{\infty }\frac{\partial }{\partial t}f_{Y_{t}}\left(y\right)\;dy-\int_{-\infty }^{\infty }\log_{e}f_{Y_{t}}\left(y\right)\;\frac{\partial }{\partial t}f_{Y_{t}}\left(y\right)\;dy \end{array}$$
  (264) $$\begin{array}{cc} \; & =-\frac{\partial }{\partial t}\int_{-\infty }^{\infty }f_{Y_{t}}\left(y\right)\;dy-\int_{-\infty }^{\infty }\log_{e}f_{Y_{t}}\left(y\right)\;\frac{\partial }{\partial t}f_{Y_{t}}\left(y\right)\;dy. \end{array}$$
  Taking another derivative, the left side of (260) becomes
  (265) $$\begin{array}{cc} \frac{d^{2}}{dt^{2}}h\left(Y_{t}\right) & =-\int_{-\infty }^{\infty }\frac{\partial^{2}}{\partial t^{2}}f_{Y_{t}}\left(y\right)\;\log_{e}f_{Y_{t}}\left(y\right)\;dy-\int_{-\infty }^{\infty }\frac{\partial }{\partial t}f_{Y_{t}}\left(y\right)\;\frac{\partial }{\partial t}\log_{e}f_{Y_{t}}\left(y\right)\;dy \end{array}$$
  (266) $$\begin{array}{cc} \; & =\int_{-\infty }^{\infty }\frac{\partial^{2}}{\partial y^{2}}f_{Y_{t}}\left(y\right)\;\log_{e}f_{Y_{t}}\left(y\right)\;dy-\int_{-\infty }^{\infty }f_{Y_{t}}^{-1}\left(y\right)\;{\left(\frac{\partial }{\partial t}f_{Y_{t}}\left(y\right)\right)}^{2}\;dy \end{array}$$
  (267) $$\begin{array}{cc} \; & =\int_{-\infty }^{\infty }\frac{\partial^{2}}{\partial y^{2}}f_{Y_{t}}\left(y\right)\;\log_{e}f_{Y_{t}}\left(y\right)\;dy-K\left(Y_{t}\right) \end{array}$$
  (268) $$\begin{array}{cc} \; & =-J\left(Y_{t}\right)-K\left(Y_{t}\right), \end{array}$$
  where
  - (265) ⟸ the first term on the right side of (264) is zero;
  - (266) ⟸ (262);
  - (267) ⟸ (258);
  - (268) ⟸ integration by parts, exactly as in [4] (or p. 673 of [19]).
       □
97. Theorem 12 reveals that the increasing function $f_{X}\left(t\right)=h\left(X+t\;V\right)$ is concave (which does not follow from the concavity of differential entropy functional of the density). In contrast, it was shown by Costa [40] that the entropy power $N\left(X+\sqrt{t}\;N\right)$, with $N∼N\;\left(0,1\right)$ is concave in *t*.

## 16. Mutual Information

98. Most of this section is devoted to an additive noise model. We begin with the simplest case in which $X_{C}$ is centered Cauchy independent of $W_{C}$, also centered Cauchy with $\varsigma (W_{C})>0$. Then, (11) yields
  (269) $$\begin{array}{cc} I(X_{C};X_{C}+W_{C}) & =h\left(X_{C}+W_{C}\right)-h\left(W_{C}\right) \end{array}$$
  (270) $$\begin{array}{cc} \; & =\log\left(4\pi (\varsigma \left(X_{C}\right)+\varsigma \left(W_{C}\right))\right)-\log\left(4\pi \varsigma (W_{C})\right) \end{array}$$
  (271) $$\begin{array}{cc} \; & =\log\left(1+\frac{\varsigma (X_{C})}{\varsigma (W_{C})}\right), \end{array}$$
  thereby establishing a pleasing parallelism with Shannon’s formula [1] for the mutual information between a Gaussian random variable and its sum with an independent Gaussian random variable. Aside from a factor of $\frac{1}{2}$, in the Cauchy case, the role of the variance is taken by the strength. Incidentally, as shown in [2], if *N* is standard exponential on (0,∞), an independent *X* on [0,∞) can be found so that X+N is exponential, in which case the formula (271) also applies because the ratio of strengths of exponentials is equal to the ratio of their means. More generally, if input and noise are independent non-centered Cauchy, their locations do not affect the mutual information, but they do affect their strengths, so, in that case, (271) holds provided that the strengths are evaluated for the centered versions of the Cauchy random variables.
99. It is instructive, as well as useful in the sequel, to obtain (271) through a more circuitous route. Since $Y_{C}=X_{C}+W_{C}$ is centered Cauchy with strength $\varsigma \left(Y_{C}\right)=\varsigma \left(X_{C}\right)+\varsigma \left(W_{C}\right)$, the *information density* (e.g., [41]) is defined as
  (272) $$\begin{array}{cc} {ı}_{X_{C};Y_{C}}\left(x;y\right) & =\log\frac{dP_{X_{C}Y_{C}}}{d(P_{X_{C}}\times P_{Y_{C}})}\left(x,y\right) \end{array}$$
  (273) $$\begin{array}{cc} \; & =\log\frac{f_{Y_{C}|X_{C}}\left(y|x\right)}{f_{Y_{C}}\left(y\right)} \end{array}$$
  (274) $$\begin{array}{cc} \; & =\log\frac{\varsigma (Y_{C})}{\varsigma (W_{C})}+\log\left(1+\frac{y^{2}}{\varsigma^{2}\left(Y_{C}\right)}\right)-\log\left(1+\frac{{\left(y-x\right)}^{2}}{\varsigma^{2}\left(W_{C}\right)}\right). \end{array}$$
  Averaging with respect to $\left(X_{C},Y_{C}\right)=\left(X_{C},X_{C}+W_{C}\right)$, we obtain
  (275) $$\begin{array}{cc} I(X_{C};Y_{C}) & =E\left[{ı}_{X_{C};Y_{C}}\left(X_{C};Y_{C}\right)\right] \end{array}$$
  (276) $$\begin{array}{cc} \; & =\log\frac{\varsigma (Y_{C})}{\varsigma (W_{C})}+\log4-\log4=\log\left(1+\frac{\varsigma (X_{C})}{\varsigma (W_{C})}\right). \end{array}$$
100. If the strengths of output Y=X+N and independent noise *N* are finite and their differential entropies are not −∞, we can obtain a general representation of the mutual information without requiring that either input or noise be Cauchy. Invoking (56) and I(X;X+N)=h(X+N)−h(N), we have
  (277) $$\begin{array}{cc} I(X;Y) & =\log\frac{N_{C}\left(Y\right)}{N_{C}\left(N\right)} \end{array}$$
  (278) $$\begin{array}{cc} \; & =\log\frac{\varsigma (Y)}{\varsigma (N)}+D\left(N\;∥\;\varsigma \left(N\right)V\right)-D\left(Y\;∥\;\varsigma \left(Y\right)V\right), \end{array}$$
  since, as we saw in (49), the finiteness of the strengths guarantees the finiteness of the relative entropies in (278). We can readily verify the alternative representation in which strength is replaced by standard deviation, and the standard Cauchy *V* is replaced by standard normal *W*:
  (279) $$\begin{array}{cc} I(X;Y) & =\frac{1}{2}\log\frac{N(Y)}{N(N)} \end{array}$$
  (280) $$\begin{array}{cc} \; & =\log\frac{\sigma (Y)}{\sigma (N)}+D\left(N\;∥\;\sigma \left(N\right)W\right)-D\left(Y\;∥\;\sigma \left(Y\right)W\right). \end{array}$$
  A byproduct of (278) is the upper bound
  (281) $$\begin{array}{cc} I(X;Y) & \leq \log\frac{\varsigma (Y)}{N_{C}\left(N\right)} \end{array}$$
  (282) $$\begin{array}{cc} \; & =\log\frac{\varsigma (Y)}{\varsigma (N)}+D\left(N\;∥\;\varsigma \left(N\right)V\right), \end{array}$$
  where (281) follows from $N_{C}\left(Y\right)\leq \varsigma \left(Y\right)$, and (282) follows by dropping the last term on the right side of (278). Note that (281) is the counterpart of the upper bound given by Shannon [1] in which the standard deviation of *Y* takes the place of the strength in the numerator, and the square root of the noise entropy power takes the place of the entropy strength in the denominator. Shannon gave his bound three years before Kullback and Leibler introduced relative entropy in [42]. The counterpart of (282) with analogous substitutions of strengths by standard deviations was given by Pinsker [43], and by Ihara [44] for continuous-time processes.
101. We proceed to investigate the maximal mutual information between the (possibly non-Cauchy) input and its additive Cauchy-noise contaminated version.
  **Theorem** **13.** *Maximal mutual information: output strength constraint.* *For any $\eta \geq \varsigma (W_{C})>0$,*
(283) $$\begin{array}{c} \max_{X:\varsigma (X+W_{C})\leq \eta }I\left(X;X+W_{C}\right)=\log\frac{\eta }{\varsigma (W_{C})}, \end{array}$$
  *where $W_{C}$ is centered Cauchy independent of X. The maximum in (283) is attained uniquely by the centered Cauchy distribution with strength $\eta -\varsigma (W_{C})$.*
  **Proof.**  For centered Cauchy noise, the upper bound in (282) simplifies to
  (284) $$\begin{array}{cc} I(X;X+W_{C}) & \leq \log\frac{\varsigma (X+W_{C})}{\varsigma (W_{C})}, \end{array}$$
  which shows ≤ in (283). If the input is centered Cauchy $X_{C}$ with strength $\eta -\varsigma (W_{C})$, then $\varsigma (X_{C}+W_{C})=\eta$, and $I(X_{C};X_{C}+W_{C})$ is equal to the right side in view of (271).
     □
102. In the information theory literature, the maximization of mutual information over the input distribution is usually carried out under a constraint on the average cost E[b(X)] for some real-valued function b. Before we investigate whether the optimization in (283) can be cast into that conventional paradigm, it is instructive to realize that the maximization of mutual information in the case of input-independent additive Gaussian noise can be viewed as one in which we allow any input such that the *output variance* is constrained, and because the output variance is the sum of input and noise variances that the familiar optimization over variance constrained inputs obtains. Likewise, in the case of additive exponential noise and random variables taking nonnegative values, if we constrain the *output mean*, automatically we are constraining the input mean. In contrast, the output strength is not equal to the sum of Cauchy noise strength and the input strength, unless the input is Cauchy. Indeed, as we saw in Theorem 1-(d), the output strength depends not only on the input strength but on the shape of its probability density function. Since the noise is Cauchy, (45) yields
  (285) $$\begin{array}{cc} \varsigma (X+W_{C})\leq \eta \; & ⟺\;\varsigma_{2,\theta }\left(X\right)\leq \varsigma \left(W_{C}\right)+\eta ,\;\;with\;\;\theta =2\log\frac{2\eta }{\eta +\varsigma (W_{C})} \end{array}$$
  (286) $$\begin{array}{cc} \; & ⟺\;E\left[\log\left(\varsigma \left(W_{C}\right)+\eta +X^{2}\right)\right]\leq 2\;\log\left(2\eta \right), \end{array}$$
  which is the same input constraint found in [45] (see also Lemma 6 in [46] and Section V in [47]) in which η affects not only the allowed expected cost but the definition of the cost function itself. If *X* is centered Cauchy with strength $\eta -\varsigma (W_{C})$, then (286) is satisfied with equality, in keeping with the fact that that input achieves the maximum in (283). Any alternative input with the same strength that produces output strength lower than or equal to η can only result in lower mutual information. However, as we saw in Item 29, we can indeed find input distributions with strength $\eta -\varsigma (W_{C})$ that can produce output strength higher than η. Can any of those input distributions achieve $I\left(X;Y\right)>\log\frac{\eta }{\varsigma (W_{C})}$? The answer is affirmative. If we let $X=V_{\beta ,2}$, defined in (9), we can verify numerically that, for β∈[0.8,1),
  (287) $$\begin{array}{c} I\left(X;X+V\right)>\log\left(\varsigma (X)+1\right). \end{array}$$
  We conclude that, at least for $\frac{\theta }{\varsigma (W_{C})}\in \left(1,\varsigma (V_{0.8,2})\right)=\left(1,3.126\ldots \right)$, the capacity–input–strength function satisfies
  (288) $$\begin{array}{c} C\left(\theta \right)=\max_{X:\varsigma (X)\leq \theta }I\left(X;X+W_{C}\right)>\log\left(1+\frac{\theta }{\varsigma (W_{C})}\right). \end{array}$$
103. Although not always acknowledged, the key step in the maximization of mutual information over the input distribution for a given random transformation is to identify the optimal *output* distribution. The results in Items 101 and 102 point out that it is mathematically more natural to impose constraints on the attributes of the observed noisy signal than on the transmitted noiseless signal. In the usual framework of power constraints, both formulations are equivalent as an increase in the gain of the receiver antenna (or a decrease in the front-end amplifier thermal noise) of κ dB has the same effect as an increase of κ dB in the gain of the transmitter antenna (or increase in the output power of the transmitted amplifier). When, as in the case of strength, both formulations lead to different solutions, it is worthwhile to recognize that what we usually view as transmitter/encoder constraints also involve receiver features.
104. Consider a multiaccess channel $Y_{i}=X_{1i}+X_{2i}+W_{i}$, where $W_{i}$ is a sequence of strength ς(W) independent centered Cauchy random variables. While the capacity region is unknown if we place individual cost or strength constraints on the transmitters, it is easily solvable if we impose an output strength constraint. In that case, the capacity region is the triangle
  (289) $$\begin{array}{c} C_{\eta }=\left\{\left(R_{1},R_{2}\right)\in {\left[0,\infty \right)}^{2}:R_{1}+R_{2}\leq \log\frac{\eta }{\varsigma (W)}\right\}, \end{array}$$
  where η>ς(W) is the output strength constraint. To see this, note (a) the corner points are achievable thanks to Theorem 13; (b) if the transmitters are synchronous, a time-sharing strategy with Cauchy distributed inputs satisfies the output strength constraint in view of (107); (c) replacing the independent encoders by a single encoder which encodes both messages would not be able to achieve higher rate sum. It is also possible to achieve (289) using the successive decoding strategy invented by Cover [48] and Wyner [49] for the Gaussian multiple-access channel: fix α∈(0,1); to achieve $R_{1}=\alpha \log\frac{\eta }{\varsigma (W)}$ and $R_{2}=\left(1-\alpha \right)\log\frac{\eta }{\varsigma (W)}$, we let the transmitters use random coding with sequences of independent Cauchy random variables with respective strengths
  (290) $$\begin{array}{cc} \varsigma_{1} & =\eta -\varsigma^{\alpha }\left(W\right)\eta^{1-\alpha }>0, \end{array}$$
  (291) $$\begin{array}{cc} \varsigma_{2} & =\varsigma^{\alpha }\left(W\right)\eta^{1-\alpha }-\varsigma \left(W\right)>0, \end{array}$$
  which abide by the output strength constraint since $\varsigma_{1}+\varsigma_{2}+\varsigma \left(W\right)=\eta$, and
  (292) $$\begin{array}{cc} R_{1} & =\log\left(1+\frac{\varsigma_{1}}{\varsigma_{2}+\varsigma \left(W\right)}\right), \end{array}$$
  (293) $$\begin{array}{cc} R_{2} & =\log\left(1+\frac{\varsigma_{2}}{\varsigma (W)}\right), \end{array}$$
  a rate-pair which is achievable by successive decoding by using a single-user decoder for user 1, which treats the codeword transmitted by user 2 as noise; upon decoding the message of user 1, it is re-encoded and subtracted from the received signal, thereby presenting a single-user decoder for user 2 with a signal devoid of any trace of user 1 (with high probability).
105. The capacity per unit energy of the additive Cauchy-noise channel $Y_{i}=X_{i}+\lambda V_{i}$, where $\left\{V_{i}\right\}$ is an independent sequence of standard Cauchy random variables, was shown in [29] to be equal to ${\left(4\lambda^{2}\right)}^{-1}\log e$, even though the capacity-cost function of such a channel is unknown. A corollary to Theorem 13 is that the capacity per unit output strength of the same channel is
  (294) $$\begin{array}{c} C_{O}=\frac{1}{\lambda }\max_{\eta \geq \lambda }\frac{\lambda }{\eta }\log\frac{\eta }{\lambda }=\frac{\log e}{\lambda \;e}. \end{array}$$
  By only considering Cauchy distributed inputs, the capacity per unit input strength is lower bounded by
  (295) $$\begin{array}{c} C_{I}\geq \max_{\gamma >0}\frac{1}{\gamma }\log\left(1+\frac{\gamma }{\lambda }\right)=\frac{\log e}{\lambda } \end{array}$$
  but is otherwise unknown as it is not encompassed by the formula in [29].
106. We turn to the scenario, dual to that in Theorem 13, in which the input is Cauchy but the noise need not be. As Shannon showed in [1], if the input is Gaussian, among all noise distributions with given second moment, independent Gaussian noise is the least favorable. Shannon showed that fact applying the entropy power inequality to the numerator on the right side of (279), and then further weakened the resulting lower bound by replacing the noise entropy power in the denominator by its variance. Taking a cue from this simple approach, we apply the entropy strength inequality (124) to (277) to obtain
  (296) $$\begin{array}{cc} I(X_{C};X_{C}+W) & =\frac{1}{2}\log\frac{N_{C}^{2}\left(Y\right)}{N_{C}^{2}\left(W\right)} \end{array}$$
  (297) $$\begin{array}{cc} \; & \geq \frac{1}{2}\log\frac{N_{C}^{2}\left(X_{C}\right)+N_{C}^{2}\left(W\right)}{N_{C}^{2}\left(W\right)} \end{array}$$
  (298) $$\begin{array}{cc} \; & =\frac{1}{2}\log\left(1+\frac{\varsigma^{2}\left(X_{C}\right)}{N_{C}^{2}\left(W\right)}\right) \end{array}$$
  (299) $$\begin{array}{cc} \; & \geq \frac{1}{2}\log\left(1+\frac{\varsigma^{2}\left(X_{C}\right)}{\varsigma_{C}^{2}\left(W\right)}\right), \end{array}$$
  where (299) follows from $N_{C}^{2}\left(W\right)\leq \varsigma_{C}^{2}\left(W\right)$. Unfortunately, unlike the case of Gaussian input, this route falls short of showing that Cauchy noise of a given strength is least favorable because the right side of (299) is strictly smaller than the Cauchy-input Cauchy-noise mutual information in (271). Evidently, while the entropy power inequality is tight for Gaussian random variables, it is not for Cauchy random variables as we observed in Item 39. For this approach to succeed showing that, under a strength constraint, the least favorable noise is centered Cauchy we would need that, if *W* is independent of standard Cauchy *V*, then $N_{C}\left(V+W\right)-N_{C}\left(W\right)\geq 1$. (See Item 119c-(a).)
107. As in Item 102, the counterpart in the Cauchy-input case is more challenging due to the fact that, unlike variance, the output strength need not be equal to the sum of input and noise strength. The next two results give lower bounds which, although achieved by Cauchy noise, do not just depend on the noise distribution through its strength.
  **Theorem** **14.** *If $X_{C}$ is centered Cauchy, independent of W with 0<ς(W)<∞, denote $Y=X_{C}+W$. Then,*
(300) $$\begin{array}{c} I\left(X_{C};X_{C}+W\right)\geq \log\frac{\varsigma (Y)}{\varsigma (W)}-\left|\log\frac{\varsigma (W)}{\varsigma \left(Y\right)-\varsigma \left(X_{C}\right)}\right|, \end{array}$$
  *with equality if W is centered Cauchy.*
  **Proof.**  Let us abbreviate $\varsigma =\varsigma \left(Y\right)-\varsigma \left(X_{C}\right)$. Consider the following chain:
  (301) $$\begin{array}{cc} D(Y\;∥\;\varsigma (Y)V)-D(W\;∥\;\varsigma (W)V)\; & =D\left(X_{C}+W\;∥\;X_{C}+\varsigma \;V\right)-D\left(W\;∥\;\varsigma \left(W\right)V\right) \end{array}$$
  (302) $$\begin{array}{cc} \; & \leq D\left(W\;∥\;\varsigma \;V\right)-D\left(W\;∥\;\varsigma \left(W\right)V\right) \end{array}$$
  (303) $$\begin{array}{cc} \; & =\log\frac{\varsigma (W)}{\varsigma }+E\left[\log\frac{\varsigma^{2}+W^{2}}{\varsigma^{2}\left(W\right)+W^{2}}\right] \end{array}$$
  (304) $$\begin{array}{cc} \; & \leq \left|\log\frac{\varsigma (W)}{\varsigma }\right|, \end{array}$$
  where
  - (301) ⟸$X_{C}$ is centered Cauchy;
  - (302) ⟸ relative entropy data processing theorem applied to a random transformation that consists of the addition of independent “noise” $X_{C}$;
  - (303) ⟸ both relative entropies are finite since ς(W)<∞;
  - (304) ⟸ the elementary observation
    (305) $$\begin{array}{c} \log\frac{\varsigma^{2}+t^{2}}{\varsigma^{2}\left(W\right)+t^{2}}\leq \left\{\begin{array}{cc} 0, & \varsigma <\varsigma (W);\\ 2\log\frac{\varsigma }{\varsigma (W)}, & \varsigma \geq \varsigma (W). \end{array}\right. \end{array}$$
  The desired bound (300) now follows in view of (278). It holds with equality in *W* being centered Cauchy as, in that case, $\varsigma \left(Y\right)=\varsigma \left(X_{C}\right)+\varsigma \left(W_{C}\right)$.    □
  Although the lower bound in Theorem 14 is achieved by a centered Cauchy, it does not rule out the existence of *W* such that $\varsigma \left(W\right)=\varsigma \left(W_{C}\right)$ and $I\left(X_{C};X_{C}+W\right)<I\left(X_{C};X_{C}+W_{C}\right)$.
108. For the following lower bound, it is advisable to assume for notational simplicity and without loss of generality that $\varsigma (X_{C})=1$. To remove that restriction, we may simply replace *W* by $\varsigma (X_{C})W$.
  **Theorem** **15.** *Let V be standard Cauchy independent of W. Then,*
(306) $$\begin{array}{c} I\left(V;V+W\right)\geq \log\left(1+\frac{1}{\lambda (W)}\right), \end{array}$$
  *where λ(W) is the solution to*
(307) $$\begin{array}{c} E\left[\log\frac{{\left(2+\lambda \right)}^{2}+W^{2}}{\lambda^{2}+W^{2}}\right]=2\;\log\left(1+\frac{1}{\lambda }\right). \end{array}$$
  *Equality holds in (306) if W is a centered Cauchy random variable, in which case, λ(W)=ς(W).*
  **Proof.**  It can be shown that, if $P_{XY}=P_{X}P_{Y|X}=P_{Y}P_{X|Y}$ and $Q_{Y|X}$ is an auxiliary random transformation such that $P_{X}Q_{Y|X}=Q_{Y}Q_{X|Y}$ where $Q_{Y}$ is the response of $Q_{Y|X}$ to $P_{X}$, then
  (308) $$\begin{array}{cc} I(X;Y) & =D(P_{X|Y}∥\;Q_{X|Y}\;\left|\;P_{Y}\right)+E[{ı}_{X;\bar{Y}}(X;Y)], \end{array}$$
  where $\left(X,Y\right)∼P_{X}P_{Y|X}$ and the information density ${ı}_{X;\bar{Y}}$ corresponds to the joint probability measure $P_{X}Q_{Y|X}$. We can participate decomposition of mutual information to the case where $P_{X}=P_{V},P_{Y|X=ₓ}=P_{W+ₓ},Q_{Y|X=ₓ}=P_{W_{c_{+x}}}$ where *W_c_* is centered Cauchy with strenght *λ* > 0. Then, $P_{X}Q_{Y|X}$ is the joint distribution of *V* and *V* + *W_C_*, and
  (309) $${ı}_{X;\bar{Y}}(x;y=\log\frac{\lambda }{1+\lambda }-\log(\lambda^{2}+{\left(y-x\right)}^{2})+\log({\left(1+\lambda \right)}^{2}+y^{2}).$$
  Taking expectation with respect to (x,y)=(V,V+t), and invoking (52), we obtain
  (310) $$E[{ı}_{X;\bar{Y}}(V;V+t)]=\log\frac{\lambda }{1+\lambda }+E[\log\frac{{\left(1+\lambda \right)}^{2}+{\left(V+t\right)}^{2}}{\lambda^{2}+t^{2}}]$$
  (311) $$=\log\frac{\lambda }{1+\lambda }+\log\frac{{\left(2+\lambda \right)}^{2}+t^{2}}{\lambda^{2}+t^{2}}.$$
  Finally, taking expectation with respect to t=W, we obtain
  (312) $$E[{ı}_{X;\bar{Y}}(V;V+W)]=E[\log\frac{{\left(2+\lambda \right)}^{2}+t^{2}}{\lambda^{2}+W^{2}}]-\log(1+\frac{1}{\lambda }).$$
  If λ=λ(W), namely, the solution to (307), then (306) follows as a result of (108). If W=ς(W)V, then the solution to (307) is λ(W)=ς(W), and the equality in (306) can be seen by specializing (271) to $\left(\varsigma \left(X_{C}\right),\varsigma \left(W_{C}\right)\right)=\left(1,\varsigma \left(W\right)\right)$.    □
109. As we just saw, if *W* is centered Cauchy, then the solution to (307) satisfies λ(W)=ς(W). On the other hand, we have
  (313) $$\begin{array}{cc} 0.302..=\varsigma (V_{2,2}) & <\lambda (V_{2,2})=0.349\ldots \end{array}$$
  (314) $$\begin{array}{cc} 4.961...=\lambda (W) & <\varsigma (W)=5.845\ldots \end{array}$$
  if *W* has the probability density function in (100).
110. As the proof indicates, at the expense of additional computation, we may sharpen the lower bound in Theorem 15 to show
  (315) $$\begin{array}{c} I\left(V;V+W\right)\geq \max_{\lambda >0}\left\{E\left[\log\frac{{\left(2+\lambda \right)}^{2}+W^{2}}{\lambda^{2}+W^{2}}\right]-\log\left(1+\frac{1}{\lambda }\right)\right\}, \end{array}$$
  which is attained at the solution to
  (316) $$\begin{array}{c} \frac{\lambda }{2+\lambda }\eta_{W^{2}}\left(\frac{1}{{\left(2+\lambda \right)}^{2}}\right)-\eta_{W^{2}}\left(\frac{1}{\lambda^{2}}\right)+\frac{1}{2\lambda +2}=0. \end{array}$$
111.
  **Theorem** **16.** *The rate–distortion function of a memoryless source whose distribution is centered Cauchy with strength ς(X) such that the time-average of the distortion strength is upper bounded by D is given by*
(317) R(D)=logς(X)D,0<D<ς(X);0,0<D≥ς(X).
  **Proof.**  If D≥ς(X), reproducing the source by (0,…,0) results in time-average of the distortion strength equal to $\frac{1}{n}\sum_{i=1}^{n}\varsigma \left(X_{i}\right)=\varsigma \left(X\right)$. Therefore, R(D)=0. If 0<D<ς(X), we proceed to determine the minimal $I(X;\hat{X})$ among all $P_{\hat{X}|X}$ such that $\varsigma (X-\hat{X})\leq D$. For any such random transformation,
  (318) $$\begin{array}{cc} I(X;\hat{X}) & =h\left(X\right)-h\left(X|\hat{X}\right) \end{array}$$
  (319) $$\begin{array}{cc} \; & =h\left(X\right)-h\left(X-\hat{X}|\hat{X}\right) \end{array}$$
  (320) $$\begin{array}{cc} \; & \geq h\left(X\right)-h\left(X-\hat{X}\right) \end{array}$$
  (321) $$\begin{array}{cc} \; & =\log\left(4\pi \varsigma (X)\right)-h\left(X-\hat{X}\right) \end{array}$$
  (322) $$\begin{array}{cc} \; & \geq \log\left(4\pi \varsigma (X)\right)-\log\left(4\pi \varsigma (X-\hat{X})\right) \end{array}$$
  (323) $$\begin{array}{cc} \; & \geq \log\frac{\varsigma (X)}{D}, \end{array}$$
  where (320) holds because conditioning cannot increase differential entropy, and (322) follows from Theorem 3 applied to $Z=X-\hat{X}$. The fact that there is an allowable $P_{\hat{X}|X}$ that achieves the lower bound with equality is best seen by letting $X=\hat{X}+Z$, where *Z* and $\hat{X}$ are independent centered Cauchy random variables with ς(Z)=D and $\varsigma \left(\hat{X}\right)=\varsigma \left(X\right)-D$. Then, $P_{\hat{X}|X}P_{X}=P_{X|\hat{X}}P_{\hat{X}}$ is such that the *X* marginal is indeed centered Cauchy with strength ς(X), and $\varsigma (X-\hat{X})=D$. Recalling (271),
  (324) $$\begin{array}{c} I\left(\hat{X};X\right)=\log\left(1+\frac{\varsigma (X)-D}{\varsigma (Z)}\right)=\log\frac{\varsigma (X)}{D}, \end{array}$$
  and the lower bound in (323) can indeed be satisfied with equality. We are not finished yet since we need to justify that the rate–distortion function is indeed
  (325) $$\begin{array}{c} R\left(D\right)=\min_{P_{\hat{X}|X}:\varsigma \left(X-\hat{X}\right)\leq D}I\left(X;\hat{X}\right), \end{array}$$
  which does not follow from the conventional memoryless lossy compression theorem with average distortion because, although the distortion measure is separable, it is not the average of a function with respect to the joint probability measure $P_{X\hat{X}}$. This departure from the conventional setting does not impact the direct part of the theorem (i.e., ≤ in (325)), but it does affect the converse and in particular the proof of the fact that the *n*-version of the right side of (325) single-letterizes. To that end, it is sufficient to show that the function of *D* on the right side of (325) is convex (e.g., see pp. 316–317 in [19]). In the conventional setting, this follows from the convexity of the mutual information in the random transformation since, with a distortion function d(·,·), we have
  (326) $$\begin{array}{c} E\left[d\left(X,{\hat{X}}_{\alpha }\right)\right]=\alpha \;E\left[d\left(X,{\hat{X}}_{1}\right)\right]+\left(1-\alpha \right)\;E\left[d\left(X,{\hat{X}}_{0}\right)\right], \end{array}$$
  where $\left(X,{\hat{X}}_{1}\right)∼P_{X}P_{\hat{X}|X}^{1}$, $\left(X,{\hat{X}}_{0}\right)∼P_{X}P_{\hat{X}|X}^{0}$, and $\left(X,{\hat{X}}_{\alpha }\right)∼\alpha P_{X}P_{\hat{X}|X}^{1}+\left(1-\alpha \right)P_{X}P_{\hat{X}|X}^{0}$. Unfortunately, as we saw in Item 35, strength is not convex on the probability measure so, in general, we cannot claim that
  (327) $$\begin{array}{c} \varsigma \left(X-{\hat{X}}_{\alpha }\right)\leq \alpha \;\varsigma \left(X-{\hat{X}}_{1}\right)+\left(1-\alpha \right)\varsigma \left(X-{\hat{X}}_{0}\right). \end{array}$$
  The way out of this quandary is to realize that (327) is only needed for those $P_{\hat{X}|X}^{0}$ and $P_{\hat{X}|X}^{1}$ that attain the minimum on the right side of (325) for different distortion bounds $D_{0}$ and $D_{1}$. As we saw earlier in this proof, those optimal random transformations are such that $X-{\hat{X}}_{0}$ and $X-{\hat{X}}_{1}$ are centered Cauchy. Fortuitously, as we noted in (107), (327) does indeed hold when we restrict attention to mixtures of centered Cauchy distributions.    □
  Theorem 16 gives another example in which the Shannon lower bound to the rate–distortion function is tight. In addition to Gaussian sources with mean–square distortion, other examples can be found in [50]. Another interesting aspect of the lossy compression of memoryless Cauchy sources under strength distortion measure is that it is optimally successively refinable in the sense of [51,52]. As in the Gaussian case, this is a simple consequence of the stability of the Cauchy distribution and the fact that the strength of the sum of independent Cauchy random variables is equal to the sum of their respective strengths (Item 27).
112. The continuity of mutual information can be shown under the following sufficient conditions
  **Theorem** **17.** *Suppose that $X_{n}$ is a sequence of real-valued random variables that vanishes in strength, Z is independent of $X_{n}$, h(Z)>−∞ and 0<ς(Z)<∞. Then,*
(328) $$\begin{array}{c} \lim_{n\to \infty }I\left(X_{n};X_{n}+Z\right)=0. \end{array}$$
  **Proof.**  Under the assumptions, h(Z)∈R. Therefore, $I\left(X_{n};X_{n}+Z\right)=h\left(X_{n}+Z\right)-h\left(Z\right)$, and (328) follows from Theorem 1-(m).    □
113. The assumption h(Z)>−∞ is not superfluous for the validity of Theorem 17 even though it was not needed in Theorem 1-(m). Suppose that *Z* is integer valued, and $X_{n}={\left(nL\right)}^{-1}\in \left(0,\frac{1}{2}\right)$ where L∈{2,3,…} has probability mass function
  (329) $$\begin{array}{c} P_{L}\left(k\right)=\frac{0.986551...}{k\;\log_{2}^{2}k},\;\;k=2,3,\ldots \end{array}$$
  Then, $I\left(X_{n};X_{n}+Z\right)=H\left(X_{n}\right)=H\left(L\right)=\infty$, while $E[|X_{n}\left|\right]=\frac{0.328289...}{n}$, and therefore, $\varsigma (X_{n})\to 0$.
114. In the case in which $V^{n}$ and $W^{n}$ are standard spherical multivariate Cauchy random variables with densities in (6), it follows from (7) that $\lambda_{X}\;V^{n}+\lambda_{W}\;W^{n}$ has the same distribution as $\left(\right|\lambda_{X}\left|+\right|\lambda_{W}\left|\right)\;V^{n}$. Therefore,
  (330) $$\begin{array}{cc} I\left(V^{n};\lambda_{X}\;V^{n}+\lambda_{W}\;W^{n}\right)\; & =h\left(\lambda_{X}\;V^{n}+\lambda_{W}\;W^{n}\right)-h\left(\lambda_{W}\;W^{n}\right) \end{array}$$
  (331) $$\begin{array}{cc} \; & =n\log\left(1+\frac{|\lambda_{X}|}{|\lambda_{W}|}\right), \end{array}$$
  where we have used the scaling law $h\left(\alpha X^{n}\right)=n\log\left|\alpha \right|+h\left(X^{n}\right)$. There is no possibility of a Cauchy-counterpart of the celebrated log-determinant formula for additive Gaussian vectors (e.g., Theorem 9.2.1 in [41]) because, as pointed out in Item 7, $\Lambda^{\frac{1}{2}}V^{n}+{\bar{\Lambda }}^{\frac{1}{2}}W^{n}$ is not distributed according to the ellipsoidal density in (8) unless Λ and $\bar{\Lambda }$ are proportional, in which case the setup reverts to that in (330).
115. To conclude this section, we leave aside additive noise models and consider the mutual information between a partition of the components of the standard spherical multivariate Cauchy density (6). If I∩J=⌀, then (17) yields
  (332) $$\begin{array}{c} I\left(\left\{V_{i},i\in I\right\};\;\left\{V_{j},j\in J\right\}\right)=h_{\left|I\right|}+h_{\left|J\right|}-h_{\left|I|+|J\right|}, \end{array}$$
  where $h_{n}$ stands for the right side of (17). For example, if i≠j, then, in nats,
  (333) $$\begin{array}{cc} I(V_{i};V_{j}) & =2\;h\left(V_{1}\right)-h\left(V_{1},V_{2}\right) \end{array}$$
  (334) $$\begin{array}{cc} \; & =2\log_{e}\left(4\pi \right)-\frac{3}{2}\left(\log_{e}\left(4\pi \right)+\gamma +\psi \left(\frac{3}{2}\right)\right)-\log_{e}\Gamma \left(\frac{3}{2}\right) \end{array}$$
  (335) $$\begin{array}{cc} \; & =\log_{e}\left(8\pi \right)-3=0.22417\ldots \end{array}$$
  More generally, the dependence index among the *n* random variables in the standard spherical multivariate Cauchy density is (see also [9,53]), in nats,
  (336) $$\begin{array}{cc} \; & D\left(P_{V^{n}}\;∥\;P_{V_{1}}\times \cdots \times P_{V_{n}}\right)=n\;h\left(V_{1}\right)-h\left(V^{n}\right) \end{array}$$
  (337) $$\begin{array}{cc} \; & =\frac{n-1}{2}\log_{e}\left(4\pi \right)+\log_{e}\Gamma \left(\frac{n+1}{2}\right)-\frac{n+1}{2}\left(\gamma +\psi \left(\frac{n+1}{2}\right)\right) \end{array}$$
  (338) $$\begin{array}{cc} \; & =\left\{\begin{array}{cc} \frac{n}{2}\log_{e}\left(8\pi \right)+\sum_{k=1}^{\frac{n}{2}}\left(\log_{e}\left(2k-1\right)-\frac{n+1}{2k-1}\right), & n\;even;\\ \frac{n-1}{2}\log_{e}\left(4\pi \right)+\sum_{k=1}^{\frac{n-1}{2}}\left(\log_{e}k-\frac{n+1}{2k}\right), & n\;odd. \end{array}\right. \end{array}$$
116. The *shared information* of *n* random variables is a generalization of mutual information introduced in [54] for deriving the fundamental limit of interactive data exchange among agents who have access to the individual components and establish a dialog to ensure that all of them find out the value of the random vector. The shared information of $X^{n}$ is defined as
  (339) $$\begin{array}{cc} S(X^{n}) & =\min_{\Pi }\frac{1}{|\Pi |-1}D\left(P_{X^{n}}\;∥\;\prod_{\ell =1}^{\left|\Pi \right|}P_{X(I_{\ell })}\right), \end{array}$$
  where $X\left(J\right)=\left\{X_{i},i\in J\right\}$, with J⊂I={1,…,n}, and the minimum is over all partitions of I:
  $$\Pi =\{I_{\ell }\neq ⌀,\ell =1,\ldots ,|\Pi |\},\;with\;\cup_{\ell =1}^{\left|\Pi \right|}I_{\ell }=I,\;I_{\ell }\cap I_{j}=⌀,\ell \neq j,$$
  such that |Π|>1. If we divide (338) by n−1, we obtain the *shared information* of *n* random variables distributed according to the standard spherical multivariate Cauchy model. This is a consequence of the following result, which is of independent interest.
  **Theorem** **18.** *If $X^{n}$ are exchangeable random variables, any subset of which have finite differential entropy, then for any partition Π of {1,…,n},*
(340) $$\begin{array}{c} \frac{1}{|\Pi |-1}D\left(P_{X^{n}}\;∥\;\prod_{\ell =1}^{\left|\Pi \right|}P_{X(I_{\ell })}\right)\geq \frac{1}{n-1}D\left(P_{X^{n}}\;∥\;P_{X_{1}}\times \cdots \times P_{X_{n}}\right). \end{array}$$
  **Proof.**  Fix any partition Π with |Π|=L∈{2,…,n−1} chunks. Denote by $n_{\ell }$ the number of chunks in Π with cardinality ℓ∈{1,…,n−1}. Therefore,
  (341) $$\begin{array}{c} \sum_{\ell =1}^{n-1}n_{\ell }=L,\;\;and\;\;\sum_{\ell =1}^{n-1}\ell \;n_{\ell }=n. \end{array}$$
  By exchangeability, any chunk of cardinality *k* has the same differential entropy, which we denote by $h_{k}$. Then,
  (342) $$\begin{array}{c} D\left(P_{X^{n}}\;∥\;\prod_{\ell =1}^{\left|\Pi \right|}P_{X(I_{\ell })}\right)=-h_{n}+\sum_{\ell =1}^{n-1}n_{\ell }\;h_{\ell }, \end{array}$$
  and the difference of the left minus the right sides of (340) multiplied by (n−1)(L−1) is readily seen to equal
  $$\begin{array}{cc} \; & -\left(n-1\right)\;h_{n}+\left(n-1\right)\sum_{\ell =1}^{n-1}n_{\ell }\;h_{\ell }+\left(L-1\right)\;h_{n}-\left(L-1\right)n\;h_{1} \end{array}$$
  (343) $$\begin{array}{cc} \; & =\left(\left(n-1\right)n_{1}-n\left(L-1\right)\right)\;h_{1}+\left(L-n\right)\;h_{n}+\left(n-1\right)\sum_{\ell =2}^{n-1}n_{\ell }\;h_{\ell } \end{array}$$
  (344) $$\begin{array}{cc} \; & \geq \left(\left(n-1\right)n_{1}-n\left(L-1\right)+\sum_{\ell =2}^{n-1}\left(n-\ell \right)n_{\ell }\right)\;h_{1}+\left(L-n+\sum_{\ell =2}^{n-1}\left(\ell -1\right)n_{\ell }\right)\;h_{n} \end{array}$$
  (345) $$\begin{array}{cc} \; & =0 \end{array}$$
  where
  - (344) ⟸ for all ℓ∈{2,…,n−1},
    (346) $$\begin{array}{c} h_{\ell }\geq \frac{\ell -1}{n-1}\;h_{n}+\frac{n-\ell }{n-1}\;h_{1}, \end{array}$$
    since $h_{1},\ldots ,h_{n}$ is a concave sequence, i.e., $2\;h_{k}\geq h_{k-1}+h_{k+1}$ as a result of the sub-modularity of differential entropy.
  - (345) ⟸ (341).
       □
  Naturally, the same proof applies to *n* discrete exchangeable random variables with finite joint entropy.

## 17. Outlook

117. We have seen that a number of key information theoretic properties pertaining to the Gaussian law are also satisfied in the Cauchy case. Conceptually, those extensions shed light on the underlying reason the conventional Gaussian results hold. Naturally, we would like to explore how far beyond the Cauchy law those results can be expanded. As far as the maximization of differential entropy is concerned, the essential step is to redefine strength tailoring it to the desired law: Fix a reference random variable *W* with probability density function $f_{W}$ and finite differential entropy h(W)∈R, and define the *W*-*strength* of a real valued random variable *Z* as
  (347) $$\begin{array}{cc} \varsigma_{W}\left(Z\right)\; & =\inf\left\{\varsigma >0:-E\left[\log f_{W}\left(\frac{Z}{\varsigma }\right)\right]\leq h\left(W\right)\right\}. \end{array}$$
  For example,
  (a) For α>0, $\varsigma_{W}\left(\alpha W\right)=\alpha$;
  (b) if *W* is standard normal, then $\varsigma_{W}^{2}\left(Z\right)=E\left[Z^{2}\right]$;
  (c) if *V* is standard Cauchy, then $\varsigma_{V}\left(Z\right)=\varsigma \left(Z\right)$;
  (d) if *W* is standard exponential, then $\varsigma_{W}\left(Z\right)=E\left[Z\right]$ if Z≥0 a.s., otherwise, $\varsigma_{W}\left(Z\right)=\infty$;
  (e) if *W* is standard (μ=1) Subbotin (108) with p>0, then, $\varsigma_{W}^{p}\left(Z\right)={E[|Z|}^{p}]$;
  (f) if *W* has the Rider distribution in (9), then $\varsigma_{W}\left(Z\right)=\varsigma_{\rho ,\theta }\left(Z\right)$ defined in (126) for θ chosen as in (110);
  (g) if *W* is uniformly distributed on [−1,1], $\varsigma_{W}\left(Z\right)=ess\sup\left|Z\right|$;
  (h) if *W* is standard Rayleigh, then $\varsigma_{W}\left(Z\right)=\inf\left\{\varsigma >0:E\left[\frac{Z^{2}}{\varsigma^{2}}-\log_{e}\frac{Z^{2}}{2\varsigma^{2}}\right]\leq 2+\gamma \right\}$ if Z≥0 a.s., otherwise, $\varsigma_{W}\left(Z\right)=\infty$.
  The pivotal Theorems 3 and 4 admit the following generalization.
  **Theorem** **19.** *Suppose h(W)∈R and ς>0. Then,*
(348) $$\begin{array}{c} \max_{Z:\varsigma_{W}\left(Z\right)\leq \varsigma }h\left(Z\right)=h\left(W\right)+\log\varsigma . \end{array}$$
  **Proof.**  Fix any *Z* in the feasible set. For any $\sigma \geq \varsigma_{W}\left(Z\right)$ such that $-E\left[\log f_{W}\left(\frac{Z}{\sigma }\right)\right]\leq h\left(W\right)$, we have
  (349) $$\begin{array}{cc} 0\leq D(\sigma^{-1}Z\;∥\;W)\; & =-h\left(Z\right)+\log\sigma -E\left[\log f_{W}\left(\frac{Z}{\sigma }\right)\right] \end{array}$$
  (350) $$\begin{array}{cc} \; & \leq -h(Z)+\log\sigma +h(W). \end{array}$$
  Therefore, $h\left(Z\right)\leq h\left(W\right)+\log\varsigma_{W}\left(Z\right)$, by definition of $\varsigma_{W}\left(Z\right)$, thereby establishing ≤ in (348). Equality holds since $\varsigma_{W}\left(\varsigma W\right)=\varsigma$.    □
  A corollary to Theorem 19 is a very general form of the Shannon lower bound for the rate–distortion function of a memoryless source *Z* such that the distortion is constrained to have *W*-strength not higher than *D*, namely,
  (351) $$\begin{array}{c} R(D)\geq h(Z)-h(W)-\log D. \end{array}$$
  Theorem 19 finds an immediate extension to the multivariate case
  (352) $$\begin{array}{c} \max_{Z^{n}:\varsigma_{W^{n}}\left(Z^{n}\right)\leq \varsigma }h\left(Z^{n}\right)=h\left(W^{n}\right)+n\;\log\varsigma , \end{array}$$
  where, for $W^{n}$ with $h(W^{n})\in R$, we have defined
  (353) $$\begin{array}{cc} \varsigma_{W^{n}}\left(Z^{n}\right)\; & =\inf\left\{\varsigma >0:-E\left[\log f_{W^{n}}\left(\varsigma^{-1}Z^{n}\right)\right]\leq h\left(W^{n}\right)\right\}. \end{array}$$
  For example, if $W^{n}$ is zero-mean multivariate Gaussian with positive definite covariance Σ, then $\varsigma_{W^{n}}^{2}\left(Z^{n}\right)=\frac{1}{n}E\left[Z^{n⊤}\Sigma^{-1}Z^{n}\right]$.
118. One aspect in which we have shown that Cauchy distributions lend themselves to simplification unavailable in the Gaussian case is the single-parametrization of their likelihood ratio, which paves the way for a slew of closed-form expressions for *f*-divergences and Rényi divergences. It would be interesting to identify other multiparameter (even just scale/location) families of distributions that enjoy the same property. To that end, it is natural, though by no means hopeful, to study various generalizations of the Cauchy distribution such as the Student-*t* random variable, or more generally, the Rider distribution in (9). The information theoretic study of general stable distributions is hampered by the fact that they are characterized by their characteristic functions (e.g., p. 164 in [55]), which so far, have not lent themselves to the determination of relative entropy or even differential entropy.
119. Although we cannot expect that the cornucopia of information theoretic results in the Gaussian case can be extended to other domains, we have been able to show that a number of those results do find counterparts in the Cauchy case. Nevertheless, much remains to be explored. To name a few,
  (a) The concavity of the entropy-strength $N_{C}\left(X+tV\right)$—a counterpart of Costa’s entropy power inequality [40] would guarantee the least favorability of Cauchy noise among all strength-constrained noises as well as the entropy strength inequality
    (354) $$\begin{array}{c} N_{C}\left(X+t\;V\right)\geq N_{C}\left(t\;V\right)+N_{C}\left(X\right). \end{array}$$
  (b) Information theoretic analyses quantifying the approach to normality in the central limit theorem are well-known (e.g., [56,57,58]). It would be interesting to explore the decrease in the relative entropy (relative to the Cauchy law) of independent sums distributed according to a law in the domain of attraction of the Cauchy distribution [55].
  (c) Since de Bruijn’s identity is one of the ancestors of the i-mmse formula of [59], and we now have a counterpart of de Bruijn’s identity for convolutions with scaled Cauchy, it is natural to wonder if there may be some sort of integral representation of the mutual information between a random variable and its noisy version contaminated by additive Cauchy noise. In this respect, note that counterparts for the i-mmse formula for models other than additive Gaussian noise have been found in [60,61,62].
  (d) Mutual information is robust against the addition of small non-Gaussian contamination in the sense that its effects are the same as if it were Gaussian [63]. The proof methods rely on Taylor series expansions that require the existence of moments. Any Cauchy counterparts (recall Item 77) would require substantially different methods.
  (e) Pinsker [41] showed that Gaussian processes are information stable imposing only very mild assumptions. The key is that, modulo a factor, the variance of the information density is upper bounded by its mean, the mutual information. Does the spherical multivariate Cauchy distribution enjoy similar properties?
112. Although not surveyed here, there are indeed a number of results in the engineering literature advocating Cauchy models in certain heavy-tailed infinite-variance scenarios (see, e.g., [45] and the references therein.) At the end, either we abide by the information theoretic maxim that “there is nothing more practical than a beautiful formula”, or we pay heed to Poisson, who after pointing out in [64] that Laplace’s proof of the central limit theorem broke down for what we now refer to as the Cauchy law, remarked that “Mais nous ne tiendrons pas compte de ce cas particulier, quil nous suffira d’avoir remarqué à cause de sa singularité, et qui ne se recontre sans doute pas dans la pratique”.

## Data Availability

Not applicable.

## Appendix A. Definite Integrals

(A1) $$\begin{array}{cc} \int_{0}^{x}\frac{1}{1+t^{2}}\;dt & =\arctan(x), \end{array}$$

(A2) $$\begin{array}{cc} \int_{-\frac{1}{2}}^{\frac{1}{2}}\log\cos\left(\pi t\right)\;dt & =\log\frac{1}{2}, \end{array}$$

(A3) $$\begin{array}{cc} \int_{-\infty }^{\infty }\frac{\log(1+t^{2})}{1+t^{2}}\;dt & =\pi \;\log4, \end{array}$$

(A4) $$\begin{array}{cc} \int_{-\infty }^{\infty }\frac{\log(\alpha^{2}-2\alpha t\cos\beta +t^{2})}{1+t^{2}}\;dt & =\pi \;\log(1+\alpha^{2}+2\alpha |\sin\beta |), \end{array}$$

(A5) $$\begin{array}{cc} \int_{-\infty }^{\infty }\frac{\log(1+t^{2})}{1+{\left(\xi t-\kappa \right)}^{2}}\;dt & =\frac{\pi }{\xi }\left(\log\left(\kappa^{2}+{\left(\xi +1\right)}^{2}\right)-2\;\log\xi \right),\;\xi >0, \end{array}$$

(A6) $$\begin{array}{cc} \kappa_{\beta ,\rho }\int_{-\infty }^{\infty }\frac{\log_{e}{\left(1+|t\right|}^{\rho })}{{\left(1+|t\right|}^{\rho }{)}^{\beta }}\;dt & =\psi \left(\beta \right)-\psi \left(\beta -\frac{1}{\rho }\right),\;\beta \;\rho >1, \end{array}$$

(A7) $$\begin{array}{cc} \int_{-\infty }^{\infty }\frac{\log_{e}\left(1+\theta^{2}t^{2}\right)}{{\left(1+t^{2}\right)}^{2}} & =\pi \left(\log_{e}\left(1+|\theta |\right)-\frac{\left|\theta \right|}{1+|\theta |}\right), \end{array}$$

(A8) $$\begin{array}{cc} \int_{-\alpha }^{\alpha }\log_{e}\left(t^{2}+\varsigma^{2}\right)\;dt & =4\varsigma \arctan\left(\frac{\alpha }{\varsigma }\right)-4\alpha +2\alpha \;\log_{e}\left(\alpha^{2}+\varsigma^{2}\right), \end{array}$$

(A9) $$\begin{array}{cc} \int_{-\infty }^{\infty }\frac{t^{2}}{{\left(1+t^{2}\right)}^{2}}\;dt & =\frac{\pi }{2}, \end{array}$$

(A10) $$\begin{array}{cc} \int_{-\infty }^{\infty }\frac{1}{{\left(1+t^{2}\right)}^{2}}\;dt & =\frac{\pi }{2}, \end{array}$$

(A11) $$\begin{array}{cc} \int_{-\infty }^{\infty }\frac{t^{2}}{{\left(1+t^{2}\right)}^{3}}\;dt & =\frac{\pi }{8}, \end{array}$$

(A12) $$\begin{array}{cc} \int_{-\infty }^{\infty }\frac{1}{{\left(\beta^{2}+t^{2}\right)}^{\nu }}\;dt\; & =\sqrt{\pi }\;\beta^{1-2\nu }\frac{\Gamma \left(\nu -\frac{1}{2}\right)}{\Gamma \left(\nu \right)},\;\nu >\frac{1}{2}, \end{array}$$

(A13) $$\begin{array}{cc} \int_{0}^{\infty }\frac{1}{{\left(1+t^{\rho }\right)}^{\nu }}\;dt\; & =\frac{\Gamma \left(\nu -\frac{1}{\rho }\right)\Gamma \left(1+\frac{1}{\rho }\right)}{\Gamma \left(\nu \right)},\;\nu >\frac{1}{\rho }>0, \end{array}$$

(A14) $$\begin{array}{cc} \int_{0}^{\pi }\log\left(\alpha +\beta \;\cos\theta \right)\;d\theta & =\pi \;\log\left(\frac{\alpha }{2}+\frac{1}{2}\sqrt{\alpha^{2}-\beta^{2}}\right),\;\alpha \geq \left|\beta \right|>0, \end{array}$$

(A15) $$\begin{array}{cc} \int_{0}^{\pi }\log{\left(\beta +\sqrt{\beta^{2}-1}\;\cos\theta \right)}^{\alpha }\;d\theta & =\pi \;P_{\alpha }\left(\beta \right),\;\beta >0, \end{array}$$

(A16) $$\begin{array}{cc} \int_{0}^{\infty }\frac{dt}{\sqrt{1+t^{2}}\;\sqrt{\beta^{2}+t^{2}}} & =K\left(\sqrt{1-\beta^{2}}\right),\;\beta \in \left(0,1\right), \end{array}$$

where

- (A2) is a special case of 4.384.21 in [24];
- (A3) is a special case of (A4);
- (A4) is 4.296.2 in [24];
- (A5) follows from (A4) by change of variable;
- (A6), with $\kappa_{\beta ,\rho }$ defined in (10) and ψ(·) denoting the digamma function, follows from 4.256 in [24] by change of variable $x={\left(1+t^{p}\right)}^{-\frac{1}{2n}}$ and n=mp;
- (A7) is a special case of 4.295.25 in [24];
- (A8) follows from 2.733.1 in [24];
- (A9)–(A10) follow from 3.252.6 in [24];
- (A11) can be obtained by integration by parts and (A10);
- (A12), with Γ(·) denoting the gamma function, is a special case of 3.251.11 in [24];
- (A13) can be obtained from 3.251.11 in [24] by change of variable;
- (A14) is 4.224.9 in [24];
- (A15) is 8.822.1 in [24] with $P_{\alpha }\left(x\right)$ the Legendre function of the first kind, which is a solution to
  (A17) $$\begin{array}{c} \frac{d}{dx}\left(\left(1-x^{2}\right)\frac{du(x)}{dx}\right)+\alpha \left(\alpha +1\right)\;u\left(x\right)=0; \end{array}$$
- (A16) is a special case of 3.152.1 in [24] with the complete elliptic integral of the first kind defined as 8.112.1 in [24], namely,
  (A18) $$\begin{array}{c} K\left(k\right)=\int_{0}^{\frac{\pi }{2}}\frac{d\alpha }{\sqrt{1-k^{2}\sin^{2}\alpha }},\;\left|k\right|<1. \end{array}$$
  Note that mathematica defines the complete elliptic integral function EllipticK such that
  (A19) $$\begin{array}{c} K\left(k\right)=\frac{\mathrm{EllipticK}\left(\frac{-k^{2}}{1-k^{2}}\right)}{\sqrt{1-k^{2}}},\;\left|k\right|<1. \end{array}$$
